# Mining RNA–Seq Data for Infections and Contaminations

**DOI:** 10.1371/journal.pone.0073071

**Published:** 2013-09-03

**Authors:** Thomas Bonfert, Gergely Csaba, Ralf Zimmer, Caroline C. Friedel

**Affiliations:** Institute for Informatics, Ludwig–Maximilians–Universität München, Munich, Germany; University of Torino, Italy

## Abstract

RNA sequencing (RNA–seq) provides novel opportunities for transcriptomic studies at nucleotide resolution, including transcriptomics of viruses or microbes infecting a cell. However, standard approaches for mapping the resulting sequencing reads generally ignore alternative sources of expression other than the host cell and are little equipped to address the problems arising from redundancies and gaps among sequenced microbe and virus genomes. We show that screening of sequencing reads for contaminations and infections can be performed easily using ContextMap, our recently developed mapping software. Based on mapping–derived statistics, mapping confidence, similarities and misidentifications (e.g. due to missing genome sequences) of species/strains can be assessed. Performance of our approach is evaluated on three real–life sequencing data sets and compared to state–of–the–art metagenomics tools. In particular, ContextMap vastly outperformed GASiC and GRAMMy in terms of runtime. In contrast to MEGAN4, it was capable of providing individual read mappings to species and resolving non–unique mappings, thus allowing the identification of misalignments caused by sequence similarities between genomes and missing genome sequences. Our study illustrates the importance and potentials of routinely mining RNA–seq experiments for infections or contaminations by microbes and viruses. By using ContextMap, gene expression of infecting agents can be analyzed and novel insights in infection processes and tumorigenesis can be obtained.

## Introduction

Next generation sequencing (NGS) technologies provide novel opportunities for transcriptomic analyses beyond simple quantification of gene expression. As one of the major challenges in analyzing RNA–seq data is the identification of the transcriptomic origin of each sequencing read (mapping), this has inspired the development of several novel RNA–seq mapping tools, e.g. TopHat [1] and TopHat2 [2], MapSplice [3], RUM [4], and RNASEQR [5]. While all of these rely on fast alignment algorithms such as Bowtie [6], they use different strategies to identify reads from exon–exon junctions, a problem unique to RNA–seq data. In general, these approaches choose the alignment with the minimum number of mismatches for each read and cannot resolve multiple possible mappings for a read with the same alignment score. This problem is addressed by our recently developed ContextMap method [7], which makes use of information provided by reads mapped to the same genomic region and likely originating from transcripts of the same gene. Thus, ContextMap does not aim at finding the mapping with the minimum number of mismatches, but the most likely mapping in the context of all other reads, in this way resolving non–unique mappings with high accuracy. It can be applied both to resolve non–unique mappings of other mapping tools as well as in standalone mode (see www.bio.ifi.lmu.de/ContextMap).

Independent of the mapping algorithm used, reads are usually only mapped against the reference genome (and sometimes transcriptome) of the species for which samples were collected. This completely ignores the possibility that reads may originate from other sources, e.g. unexpected contamination of samples, such as *Mycoplasma* species which are often found as contaminants in cell cultures, as well as viral or microbial infections of patients from which samples were derived. As RNA–seq protocols cannot distinguish between RNA from different species, mRNA from the infecting species will automatically also be sequenced. Indeed, dual RNA–seq of a pathogen and its host has recently been proposed for studying expression changes in both species simultaneously [8] and has already been performed for MCMV infection [9]. While in this case the infecting species is known and an additional mapping against the corresponding genome is sufficient, for most applications contaminations or infections are not known beforehand.

Such an application would be the diagnostic screening of patient samples for unknown microbial or viral infections. Here, precise identification of the infecting agent is essential for medical treatment. Furthermore, it can provide novel insights into diseases, in particular tumorigenesis, by connecting them to otherwise undetected infections. One example that shows this nicely are the cervical cancer–derived HeLa cells. Human papillomaviruses (HPV), in particular HPV–16 and –18, have since been recognized as a predominant cause of cervical cancer [10], [11] and HeLa cells have been shown to express transcripts of the integrated HPV–18 genome [12]. As we show in this study, HPV–18 expression can be easily detected in RNA–seq data of HeLa cells. While in this case this only confirms previous knowledge, in other cases novel connections between viral infections and tumorigenesis can be detected. For instance, Castellarin *et al.* [13] used RNA–seq of tumor and normal tissue samples to link colorectal carcinoma to *Fusobacterium* infection.

With standard RNA–seq mapping tools, mapping both against the host reference genome and all available microbial and viral genomes is only possible using a sequential approach [14] and requires additional steps for resolving non–unique read mappings that often occur due to local or global similarities between genomes. In contrast, ContextMap can be directly applied to automatically mine for reads from an arbitrary number of genomes since it already implements sophisticated strategies for resolving multiple read alignments. This makes it possible to also apply ContextMap for metatranscriptomics of species communities, e.g. the gut microbiome. While a number of such metatranscriptomics studies have already been performed [15]–[18], these generally used BLAST to identify the involved species and did not even use existing metagenomics methods (e.g. MEGAN4 [19], GRAMMy [20], or GASiC [21]) for species identification.

In this study, we show how ContextMap can be easily used to identify reads from multiple sources in parallel such as viral and microbial genomes. Furthermore, we present methods based on mapping–derived statistics to assess confidence of mappings to the identified species/strains and identify false positive hits due to similarities between genomes and missing genome sequences. While some of these methods require information only provided by the ContextMap algorithm, they can in general also be extended to post–process output of other mapping approaches. We illustrate the performance of the proposed methods on three applications. First, we use RNA–seq data of HeLa cells to characterize HPV–18 expression in these cells and correlate this to ongoing cell proliferation. Second, we illustrate the potential pitfalls of misidentifying species or strains in case of missing genome sequences based on a re–analysis of the Castellarin *et al.* data and show how these pitfalls can be avoided. Finally, for in–vitro sequencing data of a microbial community, we show how the involved species/strains can be identified despite the presence of several very closely related species/strains in the reference set and compare our results to MEGAN4, GRAMMy and GASiC as well as a number of other metagenomics tools.

## Materials and Methods

### Identifying Sequencing Reads from Multiple Sources using ContextMap

The original implementation of ContextMap presented previously [7] focused on refining initial mappings provided by other mapping algorithms. We recently developed a standalone version that can provide these initial mappings itself (see www.bio.ifi.lmu.de/ContextMap for details). Here, the central concept of both ContextMap versions is the so–called read *context*. This is defined as a set of reads originating from the same stretch of the genome, indicating that these reads were derived from the same transcript or different transcripts of the same gene. These contexts are defined based on the initial mapping and then extended in a subsequent re–alignment step, allowing a high degree of ambiguity both between and within contexts. For this purpose, ContextMap uses a modified version of Bowtie to identify spliced read alignments in a combination of forward and backward alignments. For each read not only the alignment with the minimum number of mismatches but any alignment to any context with at most a maximum number of mismatches is investigated. The unique mapping for the read to only one context is then determined by first finding the best mapping for the read in each context and subsequently finding the best context. For this purpose, a support score is used, taking into account the number of reads mapping within and around the region to which the read is aligned. Until the final step, contexts are treated independently of each other (see Figure S1).

As we show in this article, the advantage of this approach is that it allows investigating many alternative sources of reads in parallel, such as rRNA sequences, which are generally not included in reference genome assemblies of higher eukaryotes, as well as viral and microbial genomes. Contexts are then identified separately for each genome including the optimal context in each genome for each read. The final step is then used to decide for each read which of these contexts in any of the genomes considered results in the best mapping.

**Figure 1:**
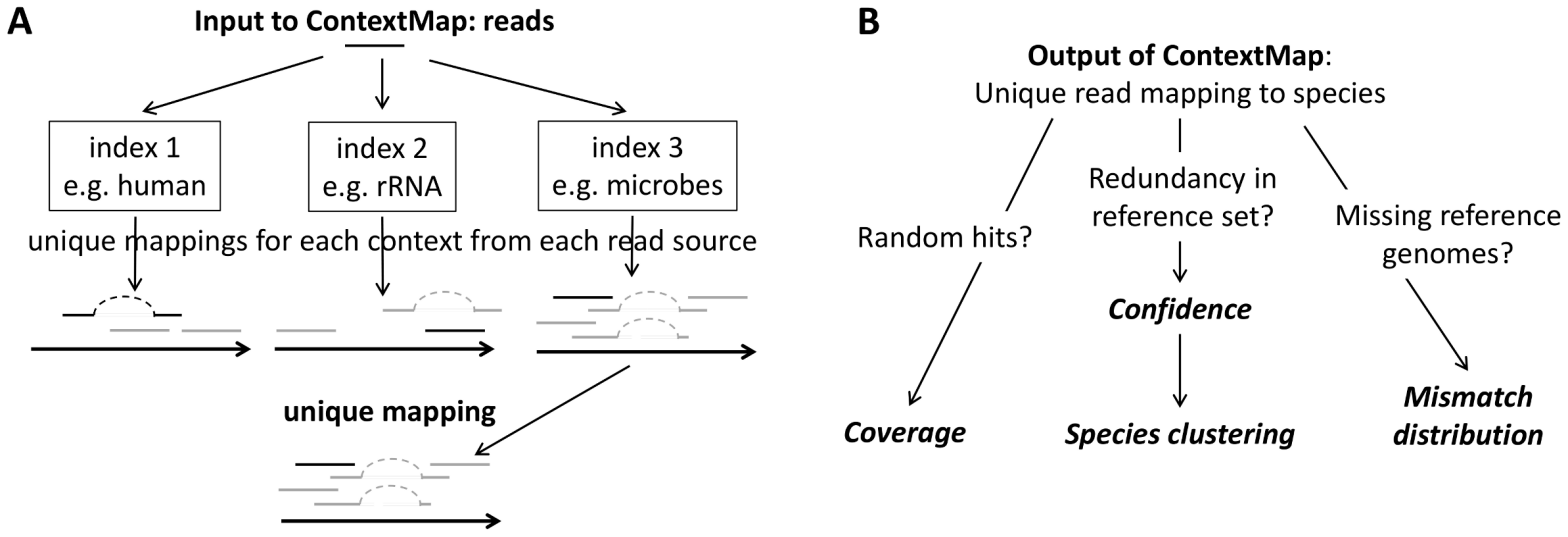
Mining RNA–seq data for infections and contaminations. (A) Approach for mapping sequencing reads in parallel to multiple sources of reads using ContextMap. (B) After obtaining unique mappings to the species in the reference set, different questions can be addressed. Random hits to only a small region of the genome can be identified by investigating coverage. Strong similarities in terms of possible read mappings between different species in the reference set can be identified by analyzing confidence and species clusterings. Finally, by analyzing mismatch distributions in terms of the Jensen–Shannon divergence, it can be determined if reads have been mapped to the correct genome or only to a close relative due to missing genome sequences or local genome similarities.

The parallel multi–species mapping is implemented by ContextMap in the following way (Figure 1 A). First, independent Bowtie indices are created for different potential read sources. Separate indices are necessary as Bowtie is limited to 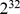
–1 characters per index. This is relevant as the human genome alone needs 73% of the maximum index size and all microbial genomes from the NCBI database taken together require 134% of the maximum index size. We, thus, generally use one index for rRNA sequences, one for the host genome, e.g. the human reference genome, one for virus genomes and two for microbe genomes. This can be easily adjusted to more indices as soon as the increasing number of sequenced virus and microbe genomes makes this necessary. After performing the initial alignment against all indices, ContextMap is then run without any further changes to define contexts, the optimal mapping for each read in each context it may belong to and finally the optimal and unique mapping for each read to any context.

In contrast to ContextMap, other RNA–seq mapping tools, which predominantly also use Bowtie, cannot be used for this application as they do not support the use of multiple indices required here due to the size and number of reference sequences and provide no way to distinguish between alternative alignments for a read to two different but related genomes with the same number of mismatches. Thus, they can only be applied sequentially by mapping first all reads e.g. against rRNA sequences, then the unmapped reads against the host reference genome, and then one microbe or virus genome one after the other. However, the latter approach also poses problems as it can lead to different results depending on the order in which genomes are mapped to in case of closely related species or strains.

### Analysis of Species Hits

The mapping of reads to reference genomes using any algorithm directly implies a set of species potentially contained in the sample. Please note that in the following we use the term species loosely, in particular in the context of misidentification of species, and it may also refer to a particular strain of a species, represented by a specific genome sequence in the reference database. In particular for bacteria, the distinction between strains and species is not clearly defined and species definition remains a difficult topic. The standard approach is now to use genome sequence differences and a cutoff of 95% average nucleotide identity is often used [22]. However, for species/strains for which no genome sequence is available, nucleotide identity to sequenced species/strains cannot be calculated. Thus, it cannot be determined whether they represent a different species or only a different strain of a species with known genome sequence.

Independent of which mapping algorithm was used to identify species potentially contained in a sample, a number of problems arise that need to be addressed. First, local similarities in the genome of one species not contained in the sample (species A) to a species contained in the sample (species B) may result in reads erroneously mapped to species A and the reporting of this species for the sample. Second, gaps in the reference database may lead to both missing and incorrect hits. If no genome from the species itself or closely related species is contained in the reference database, fast mapping algorithms, including ContextMap, which tolerate only a limited number of sequence differences, will fail to align the corresponding reads. This type of missing species hits is only a minor problem as a slower but more permissive BLAST run applied to unmapped reads may at least detect the infection by identifying more distant relatives of the infecting pathogen. A more severe problem are misalignments in case that genome sequences are only available for closely related species. In this case, reads are incorrectly aligned to these related species, resulting in the identification of wrong species. For instance, in the recent study by Castellarin *et al.* [13] several *Pseudomonas syringae* strains, which are plant pathogens, were likely misidentified in samples of colorectal carcinoma.

In the following, several statistics derived from read mappings are described that can be used to address the described problems and confidently identify the species contained in the sample (see Figure 1 B). Coverage and divergence of mismatch distributions can be calculated based on mappings provided by any algorithm. Calculation of species mapping confidence and distances between species relies on the support score calculated by ContextMap for each read mapping, but can be adapted to methods evaluating only the number of mismatches. All methods are available as part of the ContextMap software suite available at http://www.bio.ifi.lmu.de/contextmap.

#### Read numbers

The standard approach for identifying the species contained in a sample based on the read mapping is to choose those species with the highest numbers of mapped reads. This is an important measure as small read numbers tend to indicate less likely matches. However, it can be misleading as local similarities to very small regions of the genome can lead to artificially high read numbers. As a consequence, we use read numbers only as one criterion for a hit and combine this with several other measures.

#### Coverage

To identify random matches, i.e. cases in which many reads are mapped to a small genome region only, we calculate the coverage of the genome by reads:
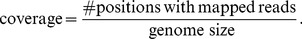
(1)


Here, only the start positions of reads are counted. Mapping of reads to only a small fraction of the genome will result in very small coverage, suggesting a random hit. However, as coverage is influenced strongly by sequencing depth, low coverage for a correct hit may be observed in case of low sequencing depth. Thus, other measures have to be used in combination with coverage.

#### Mismatch distributions

Assuming that the average sequencing error is approximately the same for all species in the sample, an increase in mismatches in aligned reads for a species indicates that the identified species differs considerably from the actual species in the sample. To identify such cases, we compare the distribution of sequencing errors on mapped reads for each predicted species hit against a reference species for which we are certain that it is contained in the sample (e.g. the host species). The difference between the two mismatch distributions is calculated using the Kullback–Leibler divergence:
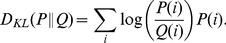
(2)


Here, 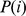 and 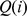 are the fractions of mapped reads with 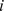 mismatches for the species under consideration and the reference species, respectively. Essentially, this quantifies the amount of information lost if 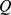 is used to approximate 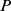. As the Kullback–Leibler divergence is non–symmetric, i.e. 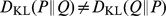, we use a symmetric measure based on 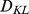, the so–called Jensen–Shannon divergence:
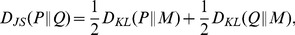
(3)where 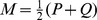. The advantage of 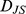 is that it is symmetric and has a clear–defined upper bound (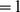 if the base 2 logarithm is used for calculating 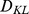 [23]). Furthermore, its square–root 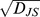 is a metric [24]. Thus, in the following we will use 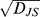 to quantify differences of mismatch distributions between the identified species and the reference genome. Please note that for our examples 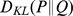 and 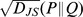 were highly correlated.

The Jensen–Shannon divergence provides a quantification of the divergence between the actual species in the sample and the identified best hit but suggests no clear cutoff to discard potential hits. Instead, the choice of the cutoff depends strongly on the application and the taxonomic level one is interested in. If the focus is on the genus level only, one may accept higher values of 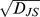 than for species identification. If one aims at identifying the actual strain even lower values of 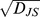 are acceptable.

As for the other measures proposed in this article, low 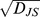 should not be considered as the only criterion for a hit as it may result from random hits to a small region of a genome with few mismatches. Thus, other measures as coverage and the species mapping confidence as introduced below should also always be evaluated. In any case, high 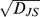 with a shift towards an increased number of mismatches indicates substantial divergence of the sequenced genome from the species in the sample, suggesting misidentification of the infecting species or strain.

#### Species mapping confidence

To identify mismappings due to similarities between genome sequences we calculate a score quantifying the confidence of read mappings to each species. Here, confidence for an individual read mapping is evaluated in terms of the support score difference between best and second–best mapping provided by ContextMap. Please note that the final output of ContextMap contains only the single best mapping for each read to any of the provided reference genome sequences. Only the score of the second–best mapping is recorded for calculation of mapping confidence. For each species 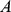, we calculate the following mapping confidence score relative to a set of other species 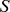 (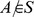):
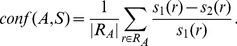
(4)where 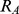 is the set of reads mapped to 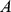, 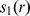 the support score for 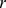 in species 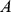, and 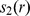 the best support score of 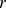 to a species in 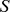. If a read 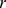 cannot be mapped at all to any other species in 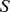, 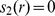. As 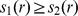, confidence is between 0 and 1 and low species confidence indicates that many of the assigned reads might alternatively be mapped to another species in 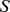 with only a little reduction in the score. The confidence score definition can be easily adapted to other mapping approaches by defining a support score measure for the corresponding mapping algorithm, e.g. based on the number of mismatches.

#### Clustering of genome hits

As ContextMap always assigns unique mappings to reads, a number of reads may still be mapped to related genomes for which they might be a better match due to sequencing errors. This is in particular the case if the genome for the microbe or virus contained in the sample is not known. In this case, reads from this microbe or virus may be dispersed over many relatives depending on local similarities. To identify such reads that likely originate from the same genome, we perform a clustering of genome hits using a dissimilarity function that is based on the relative mapping confidence of two genomes with regard to each other as defined in equation 4. The mapping dissimilarity of genome 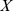 and 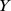 is defined as
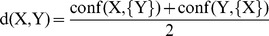
(5)


Thus, if many reads mapped to genome 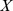 could alternatively be mapped almost as well to genome 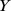 and vice versa, 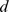 is small. Like the confidence function, 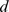 is in the range of 0 and 1. Furthermore, it is symmetric and can be used with standard distance–based clustering methods.

### Data Sets

#### RNA–seq of HeLa cells

RNA–seq data of HeLa cells were taken from the study of Guo *et al.* [25] who analyzed regulation of mammalian cells by miRNAs using both RNA–seq and ribosome profiling (Gene Expression Omnibus accession no. GSE22004). In this study, Illumina RNA–seq was performed for miRNA transfected HeLa cells at 12 and 32 h post–transfection. We used the RNA–seq data of mock and miR–155 transfected cells at 12 h post–transfection (28,735,355 and 29,595,334 36 bp reads, respectively).

#### RNA–seq of human colorectal carcinoma samples

For the second analysis, we used RNA–seq data for matched pairs of colorectal carcinoma and adjacent normal tissue samples from the study of Castellarin *et al.* [13]. Sequencing reads (75 bp) for 12 pairs of tumor and normal tissue were downloaded from the NCBI Sequence Read Archive (accession no. SRP007584). Although Castellarin *et al.* reported only the analysis of 11 sample pairs, 12 were available for download and no indication was given which of these were analyzed. Thus, we used all of them.

#### DNA–seq of *in–vitro* microbial communities

To compare our approach against standard metagenomics tools, we used pyrosequencing data of an *in–vitro* simulated microbial community [26]. In this study, cultures for 10 species (yeast, *Halobacterium sp. NRC–1, Pediococcus pentosaceous, Lactobacillus brevis, Lactobacillus casei, Lactococcus lactis subsp. cremoris SK11, Lactococcus lactis subsp. cremoris IL1403, Myxococcus xanthus DK 1622, Shewanella amazonensis SB2B, Acidothermus cellulolyticus 11B*) were grown, cell pellets from a known number of cells for each species were mixed and DNA was extracted and sequenced. Thus, the exact species contained in this sample were known beforehand. Sequencing reads for pyrosequencing data were downloaded from the NCBI Short Read Archive (accession no. SRA010765.1). To simulate NGS data, which in contrast to pyrosequencing data is characterized by both a uniform read length as well as shorter reads, we trimmed reads to 100 bp and discarded reads shorter than 100 bp, resulting in 484,629 reads.

#### Reference genomes

Reference genomes for human (GRCh37) and yeast (sacCer3) were downloaded from the UCSC genome website (http://genome.ucsc.edu/). Completed microbe and virus genomes from RefSeq release 52 were downloaded from the NCBI ftp site (2919 microbial and 4092 viral genomes). For the analysis of the colorectal carcinoma data, we additionally used draft genome sequences from the Human Microbiome Project [27].

## Results and Discussion

### HPV–18 Expression in HeLa Cells

RNA–seq data of HeLa cells from the study of Guo *et al.* [25] were mapped using ContextMap against indices for human, viral and microbe genomes and human rRNA. For the initial Bowtie runs a seed of 25 bp was used allowing up to 1 mismatch in the seed, the same settings used by Guo *et al*. In total, 5 mismatches were allowed, resulting in 11,040,798 (38.4%) and 10,162,289 (34.3%) mapped reads for the mock and miR–155 transfected cells, respectively. This is only 0.4 and 1.8 million reads less than mapped by Guo *et al.*, although they allowed an arbitrary number of mismatches outside the seed, i.e. up to 12 mismatches. Although Guo *et al.* did not perform alignment against viral or microbial genomes (but also rRNA), only few (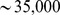) of the reads additionally mapped by ContextMap originated from viral or microbial genomes. Most reads additionally aligned by ContextMap were discarded by Guo *et al.* due to non–unique alignments. Interestingly, 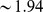 million reads originated from rRNA, which illustrates the importance of including rRNA sequences in the mapping process even though poly–A selection was performed.

**Figure 2:**
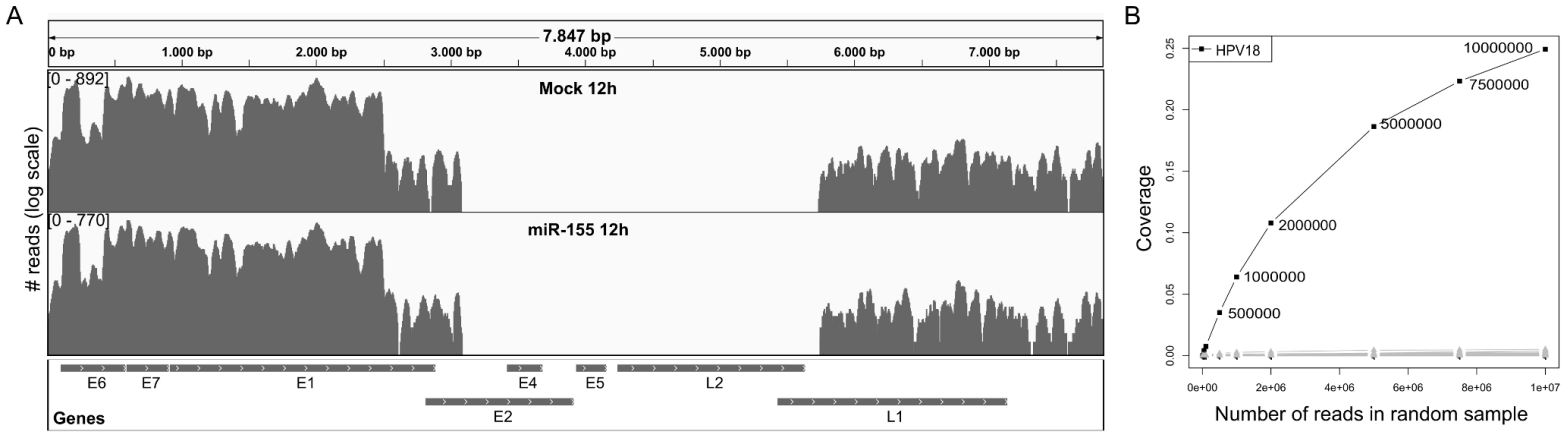
Characterization of HPV–18 infection in HeLa cells. (A) Distribution of reads across the HPV–18 genome for the mock and miR–155 transfected cells. Read numbers are shown in log scale. Expressed genes include E1 as well as E6 and E7, which are required for ongoing proliferation in cervical carcinoma [28]. L1 also appeared to be weakly expressed, however the expression pattern did not exactly correspond to the annotated gene coordinates. While the start of the gene was not expressed, L1 expression was extended to a region downstream of the gene. (B) Coverage as a function of increasing sequencing depth was evaluated by randomly sampling from the miR–155 data set. Coverage is shown as an average of ten repeated samplings for HPV–18 (black) and other species (gray). Sample size is annotated to the HPV–18 data points.

Tables S1 and S2 show coverage, mapping confidence and 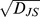 compared to the human reference genome for all species with at least 1,000 mapped reads. Figure S2 illustrates coverage for all microbial or virus hits. Here, HPV–18 is the only virus or microbe with a coverage 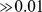 (0.34–0.37), high confidence (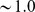) and small 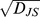 (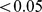) in both samples. This confirms previous reports of HPV–18 expression in HeLa cells [12]. In contrast, no reads were mapped to HPV–16, which is not expressed in HeLa cells. Figure 2 A shows the distribution of reads across the HPV–18 genome both in the mock and miR–155 transfected cells. Here, results were highly reproducible between the two samples with peaks in read heights at the same genomic locations. The mapping to genes showed that only the E6, E7 and E1 genes were strongly expressed. In addition, weaker expression by an order of magnitude was observed for L1 as well as for a region covering the end of E1 and the start of E2. However, as no reads were observed for the rest of E2, it is likely not expressed. The same was true for genes E4, E5 and L2. These observations are in accordance with recent results showing that the oncogenes E6 and E7 are essential for continued proliferation in cervical carcinoma [28]. Both genes are transcriptionally repressed by the E2 protein and loss of E2 expression leads to upregulation of E6 and E7 [29]. Thus, loss of E2 expression in HeLa cells as well as high E6 and E7 expression is consistent with their origin from cervical carcinoma cells and ongoing proliferation.

This shows that our approach is capable of identifying HPV–18 infection in HeLa cells and distinguishing this from spurious matches to other species. However, as less than 1% of reads in our samples originated from HPV–18 (22,105 and 18,491, respectively), the question remains which sequencing depth is necessary for confidently identifying such an infection. To investigate this question, we randomly sampled reads from the miR–155 data set with sample sizes between 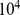 and 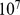 (see Figure 2 B). For each sample size, 10 random repetitions were performed and reads were mapped using ContextMap as described. Here, a sequencing depth of as low as 500,000 reads (1.7% of all reads) was sufficient to clearly distinguish the HPV–18 infection from spurious hits to other species. Although only 303 HPV–18 reads were identified on average at this sample size, almost all of these reads (90%) were mapped to distinct genome positions, resulting in a coverage of 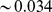. Although this coverage is small, it is more than an order of magnitude larger than for any of the other species at this sequencing depth and increases much faster with increasing sequencing depth.

To compare the proposed method against alternative approaches, we performed megablast alignments for the miR–155 data set against all microbial and viral genomes as well as human rRNA sequences and the human mitochondrial genome. Alignments with an E–value 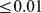 were then evaluated using MEGAN4. A megablast comparison against the complete human genome was aborted as output files already reached 10GB after mapping only 28% of reads against 30% of the genome, which would have resulted in an estimated 120GB of output (for an input of only 0.84GB). Since GASiC and GRAMMy could only be run in reasonable time on the 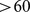
–fold smaller *in–vitro* microbial community data set by restricting them to the 10 species in question, we did not evaluate them here. MEGAN4 results are shown in Figure S3 both with and without the additional alignment against human rRNA and mitochondrial genome sequences. In both cases, HPV–18 is clearly detected although 762 fewer reads (4.1%) are assigned than by ContextMap despite the fact that an arbitrary number of mismatches and gaps are allowed by BLAST.

However, without additionally BLASTing against human rRNA and the mitochondrial genome, 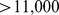 reads each are assigned to one bacterial (*Rickettsia rickettsii str. Hino*) and one viral (*Choristoneura occidentalis granulovirus*) species. When including human sequences for mapping, most of these are assigned to the inner nodes “cellular organisms” and “root”, reflecting sequence similarities between human rRNA and the *Rickettsia* genome (lowest common ancestor (LCA) = “cellular organisms”) and the human mitochondrial genome and the *Choristoneura* genome (LCA = “root”), respectively. These results show the importance of also including the host species into mapping, as otherwise *Rickettsia* and *Choristoneura* would be reported erroneously for this sample. Here, MEGAN4 provides no direct way for identifying these hits as suspicious, e.g. by calculating coverage or mismatch distributions, or for resolving the non–uniquely mapped reads assigned to inner nodes. In contrast, ContextMap correctly assigns 90% of the *Choristoneura* BLAST hits to human rRNA and only 5% to *Choristoneura*. Furthermore, 88% of the *Rickettsia* BLAST hits are correctly identified as originating from human RNA (83% from mitochondrial RNA) by ContextMap and only 1% are assigned to *Rickettsia*. In addition, the few *Choristoneura* and *Rickettsia* reads assigned by ContextMap are clearly flagged as misalignments by very high values of 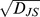 (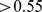).

### The Microbiome of Colorectal Carcinoma

In the second analysis, we focused on the RNA–seq data of colorectal carcinoma and adjacent normal tissue from the study of Castellarin *et al.* [13]. This data set was interesting as they identified a *Fusobacterium* to be enriched in colorectal carcinoma cancer. In addition, they reported a number of microbes that are unlikely to occur in colon tissue, e.g. *Pseudomonas fluorescens SBW25*, which was found at high levels in all samples, and two *Pseudomonas syringae* strains. *P. fluorescens* is mostly found in soil and water, whereas *P. syringae* are plant pathogens. While in the first case occurrence in colon samples might still be possible, e.g. due to contamination, in the latter case it is very unlikely. Although mapping with ContextMap also identified all three *Pseudomonas* species in all tumor and normal tissue samples, 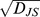 compared to the human reference genome was larger than 0.2 in all three cases (Figure S4), in particular for *Pseudomonas syringae pv. syringae* where more than half of the reads had at least 3 mismatches (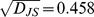). This indicates that the actual *Pseudomonas* species contained in the sample is not yet sequenced, resulting in reads from these species mapped to a number of related *Pseudomonas* species.

Based on these observations, we performed the same analysis for *Fusobacterium*. Previously, Castellarin *et al.* identified *Fusobacterium nucleatum subsp. nucleatum* as overrepresented in the RNA–seq data of the tumor tissues. Subsequent DNA sequencing of a *Fusobacterium* culture isolated from the tumor samples and mapping of reads against additional *Fusobacterium* draft genomes from the Human Microbiome Project (HMP), however identified *Fusobacterium sp. 3_1_36A2* as a much better match than *F. nucleatum*. As *F. sp. 3_1_36A2* was extracted from the colon of a patient, this makes more sense than *F. nucleatum*, which was isolated from the human oral cavity and is most commonly found there.

**Figure 3:**
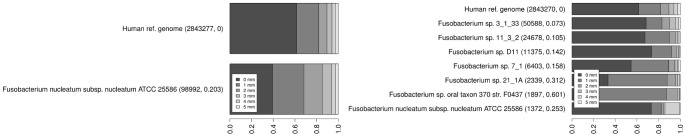
Comparison of mismatch (mm) distributions for *Fusobacteria* . Results are shown for species identified by ContextMap with at least 20 reads on the colorectal carcinoma samples for patient 1 using either only the completed microbe genomes as reference set (left) or also the human microbiome draft genome sequences (right). Distributions are compared against the average mismatch distribution for the human genome. Number of reads mapped to each genome and 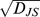 are indicated in parentheses.

To re–capitulate their analysis, we performed mapping of tumor samples using ContextMap both with and without the human microbiome in addition to the RefSeq genomes. Without the human microbiome index, *F. nucleatum* was identified in all tumor samples, in particular in samples from patient 1 (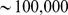 reads, Figure 3). However, the mismatch distribution differed considerably from the mismatch distribution for the human genome (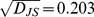 for patient 1), clearly indicating that *F. nucleatum subsp. nucleatum* is not contained in the sample but only a related species. Indeed, when performing mapping including the human microbiome, almost all of the reads originally mapped to *F. nucleatum* are mapped to contigs of other *Fusobacteria* species, such as *sp. 3_1_33, sp. 11_3_2, sp. D11, sp. 7_1*, and *sp. 21_1A*, which were isolated from biopsy tissues from the gastrointestinal tract. Furthermore, the primer sequences used by Castellarin *et al.* to confirm the presence of *Fusobacterium* are a better match to these species, with both primers matching with at most 2 mismatches, whereas for *Fusobacterium nucleatum* one primer has 3 mismatches.

Among the identified *Fusobacteria*, *F. sp. 3_1_33* has the highest number of reads for patient 1 (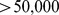) and smallest 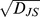 (0.073). It is also enriched in the tumor sample compared to the normal tissue, but not as strongly as some other species from the HMP with fewer mapped reads, in particular some *E. coli* strains (Table S3). Although a comparatively small number of reads (1,372) are still assigned to *F. nucleatum* even with the inclusion of the human microbiome, the mismatch distribution still diverges strongly from the human reference (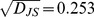) and is unusual in that it has a higher number of reads both with zero and with four mismatches. Together with the observations that 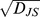 for *F. sp. 3_1_33* is still higher than in the HPV–18 example, a number of other *Fusobacteria* are also found with substantial read numbers, and most of the *F. sp. 3_1_33* reads can be aligned almost equally well to the other gastrointestinal *Fusobacteria*, this suggests that *F. sp. 3_1_33* is also not the actual strain in the sample. However, it appears to be a much closer relative than *F. nucleatum*. This also shows that 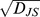 should always be analyzed in combination with read numbers as local similarities may allow the mapping of some reads to a wrong species with few mismatches.

To compare our results against other approaches, we extracted all reads for the tumor sample of patient 1 that were not mapped to human sequences (including rRNA) by ContextMap (404,234 reads) and performed both megablast and novoalign (http://www.novocraft.com) alignments for these reads against virus and microbe genomes and the human microbiome. Novoalign was used by Castellarin *et al.* to align reads to the bacterial and viral genomes after filtering out all reads that could be aligned to human rRNA, cDNA or the reference genome using BWA [30], a fast short read aligner applying a similar strategy as Bowtie. Thus, we effectively recapitulated their analysis in our study, this time also including the human microbiome. Again MEGAN4 was applied to the BLAST output as shown in Figure S5. Almost all (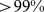) of the *Fusobacteria* reads could be aligned to more than one *Fusobacterium*, thus, resulting in an assignment of these reads to their LCA by MEGAN4. In addition, MEGAN4 allows no further analysis as to which of the identified *Fusobacteria* is the most likely candidate or closest relative of the species or strain contained in the sample.

**Figure 4:**
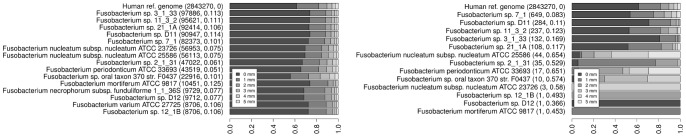
Number of reads and mismatch distributions for the novoalign mapping on the *Fusobacteria*. Results are shown for species identified by aligning with novoalign against viral and microbial genomes and the human microbiome for the patient 1 colorectal carcinoma sample. Only reads were used that were not mapped to human sequences by ContextMap. Mismatch distributions are compared against the average mismatch distribution for the human genome derived from the ContextMap mapping. Number of reads mapped to each genome and 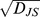 are indicated in parentheses. The left–hand side shows results if multiple read alignments with the same maximum score to different species are allowed. The right–hand side shows the results for unique alignments only.

Novoalign was applied in two modes: one outputting all alignments for a read with the same maximum score and one outputting only unique alignments (Figure 4). To compare against the ContextMap results and calculate 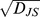 compared to the reads mapped to human by ContextMap, we then extracted only those alignments with at most 5 mismatches and no gaps. Please note that read numbers were hardly increased for the *Fusobacteria* if gaps were allowed. For evaluation of the novoalign mode allowing multiple alignments, we used only one of the best alignments for each read for each genome, but allowed multiple alignments with equal score to different genomes. Here, almost all reads could be aligned equally well to more than one genome with 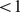
% unique read alignments per genome. Although 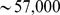 reads were still aligned to *F. nucleatum*, only 44 of these were unique and 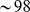
% were aligned equally well to *F. sp. 3_1_33*. Again, this illustrates the problems similarities between sequenced genomes present for mapping algorithms that are based only on the individual read alignments. Without taking into account alignments of other reads, they may only either completely exclude or include non–unique alignments. In this application, a restriction to unique alignments would vastly underestimate *Fusobacterium* expression in the sample, whereas the inclusion of non–unique mappings would result in the reporting of essentially all of the identified *Fusobacterium* species. In this case, evaluation of 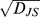 is not meaningful as due to the multiple mappings the sets of reads assigned to each species and, consequently, the calculated mismatch distributions are very similar.

### Meta–transcriptomics for an in–vitro Simulated Microbial Community

For the final analysis, we analyzed DNA sequencing data for an *in–vitro* simulated microbial community by Morgan *et al.* [26] and compared our results against several state–of–the–art metagenomics tools, in particular MEGAN4 [19], GRAMMy [20], and GASiC [21]. This data set was selected as the species contained in the samples were known. Furthermore, it constituted a challenging application due to strong similarities of the genomes of the microbial strains contained in the sample to other sequenced genomes. One example for this is *Halobacterium sp. NRC–1*, whose genome is almost identical to the *Halobacterium salinarum R1* genome [31]. They differ only by 4 base changes, 5 single–nucleotide indels and 3 longer indels between 133 and 10,007 bp long.

We investigated the performance of ContextMap on this data set using a reference containing the yeast genome and all microbial and viral genomes downloaded from NCBI (see methods) and allowing 5 mismatches. To compare our results against BLAST as well as MEGAN4 and GRAMMy, which use BLAST alignments as input, we performed megablast searches against the same genomes and extracted all alignments with the maximum score for each read, using only alignments without gaps and at most 5 mismatches. Here, 12% of reads could be aligned equally well to at least two different RefSeq entries using BLAST. In addition, we applied GASiC to all genomes from the same genus as any of the species contained in the sample (122 RefSeq entries, 92 taxa). The same restriction was applied to GRAMMy as both methods already took more than 7 CPU hours on this smaller set compared to 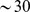 min for ContextMap on all microbes and viruses (Table S4).

Table S5 lists the microbe and virus hits identified by ContextMap with a coverage 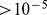 and at least 20 reads. Here, ContextMap identified all of the microbial species contained in the sample, but also several related strains and prophages. As *Myxococcus xanthus* had the highest number of mapped reads, we used it as reference for calculating 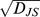. Interestingly, all microbes that are contained in the sample had a higher mapping confidence than all other hits despite low numbers of reads for some of them.

**Figure 5:**
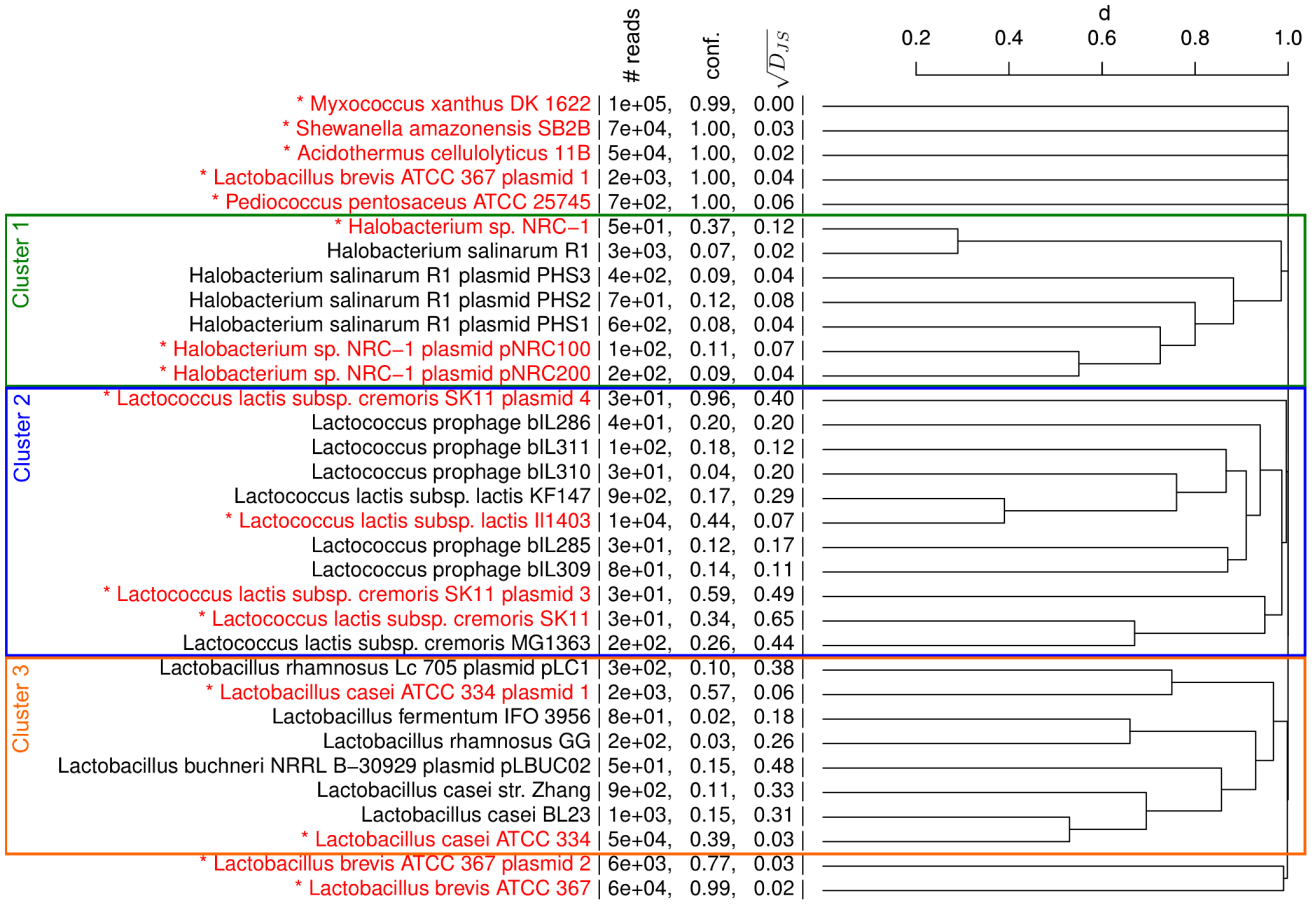
Hierarchical clustering (average linkage) of microbes and viruses . Results are shown for hits with a coverage 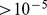 and at least 20 mapped reads as determined by ContextMap. Microbes actually contained in the sample are indicated in red and by an asterisk and the three clusters discussed in the text are marked by rectangles. In addition, number of reads, confidence and 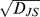 are indicated next to the microbe names.

For five species, identification is straightforward based on this list. *A. cellulolyticus*, *S. amazonensis*, *L. brevis*, and *M. xanthus* are characterized by high mapping confidence (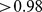), low 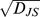 (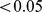) and high number of reads and coverage. For *P. pentosaceus* confidence is also high and 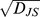 still relatively low (0.064), but coverage is quite small (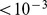). However, as 90% of the reads map to distinct positions, it is clearly a correct hit and the low coverage is likely due to low abundance of *P. pentosaceus* in the simulated community. In the clustering of species hits, these five species also form distinct clusters with no similarities to any of the other species hits (Figure 5).

For the remaining hits the situation is less clear–cut. Here, clustering identified three large groups among these: (1) a *Halobacterium* cluster, (2) a *Lactococcus* cluster, and (3) a *Lactobacillus* cluster. In the first case, *H. sp. NRC–1* clusters tightly with *H. salinarum R1* and the plasmids also cluster together, reflecting the small number of sequence differences between these. In all of these cases, 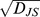 is relatively small (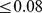), with the only exception being *H. sp. NRC–1* for which all 50 reads have zero mismatches, i.e. fewer mismatches on average (Figure S6). Additionally, confidence for *NRC–1* (0.37) is much higher than for *R1* (0.07). Thus, although significantly fewer reads are mapped to *NRC–1*, all other mapping statistics support the presence of the *NRC–1* strain rather than the *R1* strain. However, since both strains are almost identical, 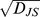 is still very low for the R1 strain (0.02).

In the second cluster, read numbers, confidence and 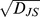 clearly indicate the presence of *L. lactis subsp. lactis Il1403*. Although 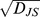 (0.07) is somewhat increased compared to the microbes identified unambiguously, it is not yet large enough to question this hit. For all other *Lactococcus lactis* strains, in particular *cremoris SK11*, the number of mismatches is significantly increased, indicating that these are not contained in the sample. This is surprising as *SK11* was part of the community. The reason for this is that 99% of the reads potentially mapping to *SK11* can be aligned equally well to other species, in particular to *L. lactis subsp. lactis Il1403*. As the latter is more abundant, it ends up with most of the reads, apart from those with too many mismatches. Finally, analysis of the last cluster confirms the presence of *L. casei ATCC 334* as it is characterized by high coverage and confidence and sufficiently low 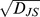 (0.06). The other strains in the cluster, in particular the *BL23* and *Zhang L. casei* strain, can be clearly excluded due to high 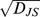 (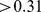) and low coverage (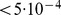) and confidence (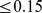).

In summary, these results show that ContextMap can be used to correctly identify all species in the community including the strain, with the exception of *cremoris SK11*. However, analysis of results for MEGAN4 (Figure S7), GASiC (Table S6) and GRAMMy (Table S7) shows that none of these identify *cremoris SK11*, at least not with more confidence than for other species/strains not contained in the community. MEGAN4 assigns almost all of the *cremoris SK11* reads to the LCA of the *cremoris* and *lactis* subspecies. GASiC assigns a p–value of 1, i.e. considers it an insignificant hit. Finally, GRAMMy, which only estimates relative abundances but performs no read mapping, assigns an abundance of 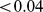
%, less than assigned to *L. lactis subsp. lactis KF147* (0.17%), which is not part of the community.

Apart from *cremoris SK11*, GASiC fails to identify *H. sp. NRC–1* and *P. pentosaceus* but otherwise predicts only microbes contained in the community. Thus, GASiC is the most restrictive of the analyzed approaches. MEGAN4 does not really resolve multiple mappings but assigns reads with multiple mappings to the LCA of these microbes. Based on the number of reads assigned uniquely to any of the children of such an LCA, the correct microbes can then be predicted. In this example, the predictions would be correct with the exception of *SK11* and *H. salinarum*, where the *NRC–1* strain cannot be properly distinguished. Nevertheless, even when combining read numbers for the microbes and the LCA, ContextMap generally identifies 2.5–6.5% more reads per microbe (including plasmids). Furthermore, assignments to inner nodes of the phylogenetic tree by MEGAN4 do not allow calculation of mismatch distributions as corresponding genome sequences are not known. Even for the leaves of the taxonomic tree, additional statistics of alignment quality or coverage are not directly accessible and can only be obtained by extracting the assigned reads and performing this analysis using additional scripts.

**Figure 6:**
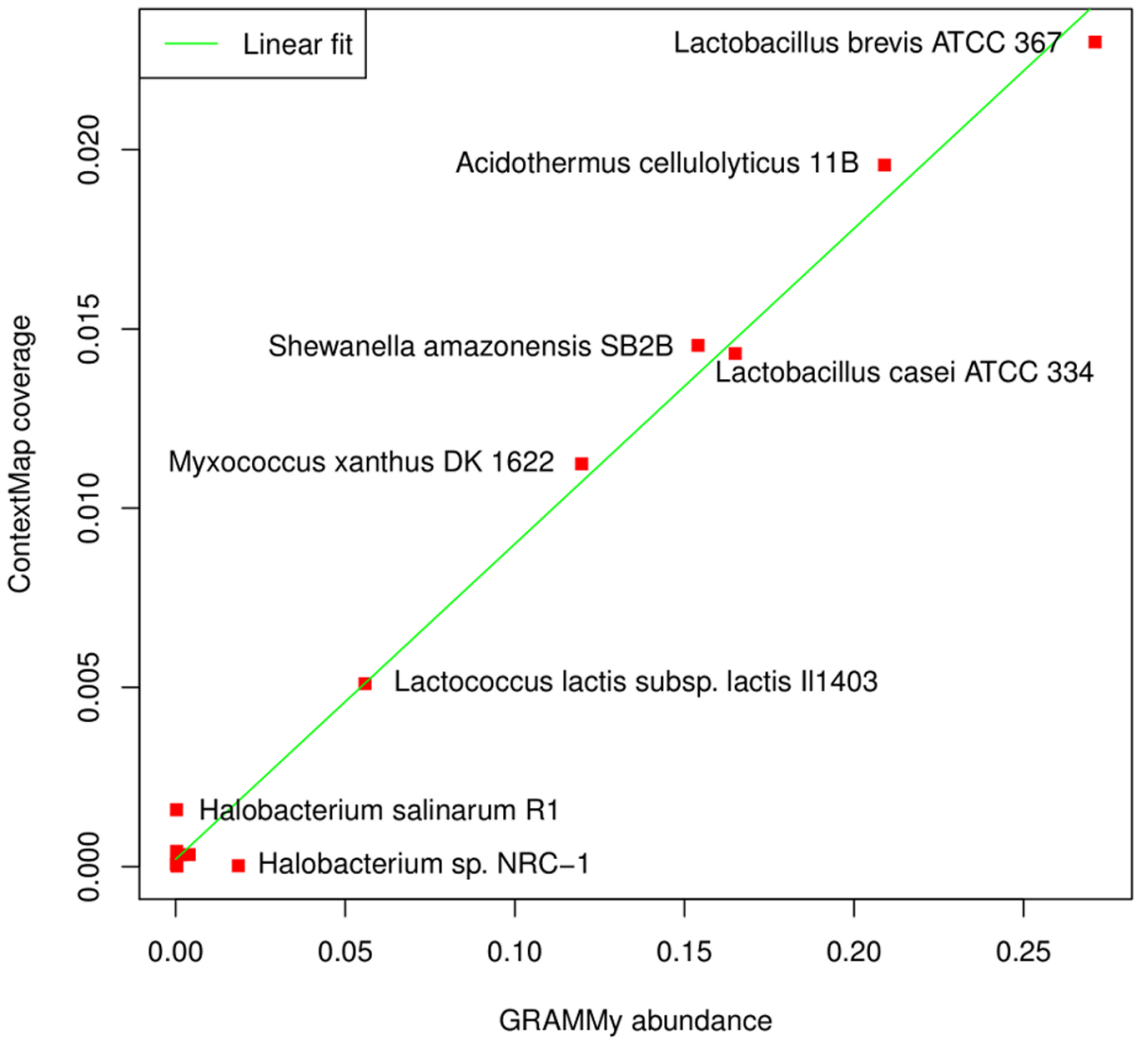
Comparison of abundance calculated by GRAMMy and coverage determined by ContextMap on the microbial community data set. Results are shown for all taxa identified by GRAMMy with a relative abundance of at least 0.1%. The green line indicates a linear fit to the data.

GRAMMy correctly identifies 7 of the 9 microbes with an estimated abundance of 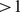
%, but also assigns a very low abundance to *P. pentosaceus* (0.4%). Remarkably, the relative frequency estimated by GRAMMy and the coverage calculated by ContextMap are highly correlated (correlation coefficient 0.995), in particular for microbes with high coverage (Figure 6). This indicates that coverage as determined by ContextMap provides a reliable estimation of the relative frequencies identified by GRAMMy. As ContextMap is much faster than GRAMMy, it can thus be used to replace GRAMMy for applications where GRAMMy is too inefficient.

We also evaluated a number of other metagenomics tools for binning/classifying sequencing reads or identifying relative abundance of species. This includes alignment–based approaches (MG–RAST [32], MetaPhyler [33], SOrt–ITEMS [34], MARTA [35], MLTreeMap [36]), composition–based approaches (PhyloPhytiaS [37], ClaMS [38], Phymm [39]) and a hybrid approach (PhymmBL [39]). Here, comparison of the results was difficult as several approaches only perform classification at the genus– (MetaPhyler) or species–level (MG–RAST, PhyloPythiaS, MARTA), but do not identify individual strains. Thus, we could not evaluate their performance in distinguishing the *Halobacterium* and *Lactococcus lactis* strains. Furthermore, only MG–RAST, MetaPhyler, Phymm and PhymmBL were developed for NGS reads as short as 100 bp, while the other tools require longer reads. Thus, the meaningfulness of the comparison against these other approaches is limited. Results for all tools are shown and discussed in Tables S8 (MG–RAST), S9 (MetaPhyler), S10 (SOrt–ITEMS), S11 (MARTA), S12 (MLTreeMap), S13 (PhyloPhytiaS), S14 (ClaMS) and S15 (Phymm and PhymmBL). In summary, although the correct species or at least genera were usually identified, performance at the level of the individual strains was usually poor as often wrong strains were ranked higher than strains contained in the community. A particular poor performance was observed for the composition–based approaches PhyloPhytiaS and ClaMS, which likely suffered from the short sequencing read length.

The analysis of runtime and memory requirements on this data set (Table S4) showed that ContextMap was both faster than almost all other approaches (apart from MetaPhyler) and required less or a comparable amount of memory (with the exception of MLTreeMap and GRAMMy if memory requirements of the BLAST run to provide the input for GRAMMy are not counted). The comparison for the other two data sets was less informative as ContextMap was either applied on more reads as in the case of the colorectal carcinoma data set or more reference sequences as in the case of the HeLa cell RNA–seq data. In the first case, ContextMap took 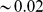 sec per read on the complete 5,343,842 read set for patient 1, whereas BLAST took 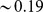 sec per read on the smaller 404,234 read set without human reads and novoalign only 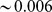 sec per read. Thus, ContextMap was much faster than BLAST but slower than novoalign. In contrast, ContextMap required much less memory with 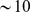
G for the complete 5 million read set compared to the 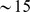
G required by novoalign for only 400 k reads and 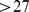
G required by BLAST. This large memory requirement (and also runtime) of BLAST for this relatively small data set was rather remarkable, in particular in comparison to the similar–sized microbe data set and the much larger HeLa data set, which both only required 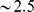
G. The reason for this is the much larger number of possible hits per read found by BLAST in the colorectal carcinoma data set (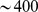 hits per read) as compared to the microbe (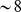 hits per read) and HeLa data set (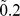 hits per read). It should be noted that in the latter case we did not perform simultaneous mapping with BLAST against the complete human genome as the estimated output size was too large. Thus, these results suggest that a complete run of BLAST on the same references as used for the ContextMap run would have resulted in substantially increased runtime and memory requirements compared to ContextMap.

## Conclusions

In contrast to microarray experiments, RNA–seq is not limited to previously defined probes but allows quantification of all transcripts in the cell, including also transcripts expressed by viral or microbial pathogens. However, current mapping approaches generally ignore the possibility of multiple origins of reads and are not designed to resolve resulting non–unique mappings. Thus, RNA–seq experiments are not routinely mined for the presence of contaminations or infections. Previous studies explicitly focusing on metatranscriptomics generally used only BLAST despite the availability of a number of metagenomics tools for identifying the species in the sample. As some of these tools were published only very recently, this might explain why they have not yet permeated the metatranscriptomics/−genomics community.

In this study, we showed how different sources of reads can be easily investigated in parallel using ContextMap without limitations to the number of potential sources investigated. This allows unbiased screening of RNA–seq data for transcripts of any species with a sequenced genome. ContextMap is particularly suited to this task as it tolerates a large degree of ambiguous mappings at intermediate steps, allowing multiple mappings to different species during these steps. These multiple mappings are then resolved in the final step using a support score calculated based on other reads aligned to the same region. From this support score, a confidence value can be calculated for each individual read mapping and the confidence of mappings for identified species and similarity of two species in terms of possible read mappings can be evaluated. This is of particular importance when mining RNA–seq data for the presence of related species. As previously published mapping methods generally cannot resolve multiple mappings and no scoring of alignments apart from mismatch counting is performed, the number of reads they cannot uniquely assign to a species is substantial. For instance, in the case of the microbial community, 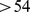
% of *L. lactis subsp. lactis Il1403* reads can be aligned equally well to other *L. lactis* subspecies and thus cannot be resolved by mapping tools relying only on alignment quality.

Our approach was evaluated first on previously published RNA–seq data sets for HeLa cells, where it allowed the identification and characterization of HPV–18 expression leading to ongoing proliferation in this cervical carcinoma–derived cell line. Here, we showed that relatively small sequencing depth can already be sufficient for reliable detection of pathogen infections, e.g. for diagnostic purposes. A comparison against BLAST combined with MEGAN4 showed the importance of aligning reads against both host and pathogen species, as local sequence similarities of microbial or viral genome sequences to human sequences, in particular rRNA and mitochondrial DNA, would otherwise lead to wrong microbial or viral hits. While ContextMap correctly resolved most of the resulting non–unique hits, MEGAN4 effectively only flagged them as non–unique hits by assigning them to internal nodes close or equal to the root of the phylogenetic tree.

A second problem arising in the context of both metatranscriptomics and metagenomics are missing genome sequences for the species/strains in the sample, which may result in misalignments of reads to related species or strains. To identify such cases, we proposed to analyze differences of mismatch distributions compared to a reference species known to be in the sample, e.g. the host species. This can be automatically evaluated using the Jensen–Shannon divergence and the usefulness of this approach was illustrated on the colorectal carcinoma data from the Castellarin *et al.* study. Here we showed that divergence of the mismatch distributions on the RNA–seq data suggested that *F. nucleatum* was not the *Fusobacterium* species in the tumor sample. Instead, a different *Fusobacterium* sequenced for the human microbiome project was identified as a more likely candidate. Again, application of MEGAN4 to BLAST results only indicated the presence of *Fusobacteria* in the sample, but could provide no further resolution as to which of the sequenced *Fusobacteria* is most likely present in the sample or most closely related to the species in the sample. We also compared our approach against the strategy used by Castellarin *et al.* by applying novoalign both to complete virus and microbe genomes and the human microbiome. Again, this approach suffered from the high similarity between *Fusobacteria*, resulting almost exclusively in non–unique hits.

Finally, we applied ContextMap to metagenomics of the *in–vitro* simulated microbial community to compare it against state–of–the–art metagenomics tools. Here, ContextMap vastly outperformed both GASiC and GRAMMy in terms of runtime, while also providing more helpful results. GASiC missed 3 of 9 microbial species in the community and furthermore allowed multiple alignments of reads to different species. In contrast, GRAMMy only determines relative abundances and does not perform any mapping of reads. Thus, it does not allow the analysis of gene expression or mismatch distributions. The latter also applies to MEGAN4, which performs no real resolution of ambiguous alignments and only assigns reads with multiple alignments to the lowest common ancestor of the corresponding species. Thus, neither GRAMMy nor MEGAN4 offer the same possibilities for gene expression analysis of microbes and viruses and identification of missing genome sequences as ContextMap, while GASiC is both much too slow and too restrictive for this application. Finally, comparison against several other metagenomics tools showed that all of these had problems in identifying the correct microbial strains contained in the sample.

Although analysis of coverage, confidence and Jensen–Shannon divergence provided by ContextMap requires some user interaction, in particular for picking thresholds, the same applies to GRAMMy, which also provides no natural cut–off on the predicted frequencies. In contrast, both GASiC and MEGAN4 basically do not allow any user interaction to fine–tune results. Despite the fact that GASiC calculates p–values, these are in most cases either 0 or 1 (at least in our application), allowing no tuning of thresholds to trade off sensitivity and specificity. Moreover, MEGAN4 provides no interface to resolve ambiguous mappings of reads assigned to an LCA or evaluate alignment quality, coverage or the other useful measures proposed here to improve the results. Finally, none of the other metagenomics tools provides any clear cutoff to determine the actual species in the sample, but only allow ranking of the possible hits, generally in terms of read numbers or estimated abundances. In any case, defining fixed thresholds for any application is likely not meaningful, as appropriate thresholds will strongly depend on the particular research question. For instance, if knowing the particular strain is of importance, e.g. in a diagnostic application where pathogenic or antibiotic–resistant strains have to be correctly identified, much lower values of Jensen–Shannon divergence would be allowed. In contrast, if only the genus or species is relevant, one might even merge species or strains into one group if they are clustered together based on the mapping similarity measure we proposed.

On alternative approach that was not evaluated in this study is PathSeq [40], a software explicitly focused on identifying microbes from sequencing data of human tissues. We did not evaluate this software as it could only be run using Amazon Web Services, thus requiring payment for using the web services and making it not available for free. However, the pipeline basically consists of a mapping of reads against human sequences first and then a mapping of unaligned reads against microbial and viral sequences using BLAST, which is similar to the BLAST approach we evaluated in this study. Thus, we expect PathSeq to encounter the same problems, i.e. high numbers of non–unique hits due to similarities between microbial and viral species, no proper resolution of non–unique hits and misidentifications in case of missing genome sequences.

Finally, it should be noted that the metrics we proposed here for evaluating potential species hits are not limited to ContextMap but can be easily extended to other mapping tools or meta–transcriptomic pipelines to further post–process their output. For coverage and divergence of mismatch distributions, this is relatively straightforward but requires a strategy to address non–unique mappings. As shown for the novoalign results on the colorectal carcinoma data, mismatch distributions are not meaningful if high numbers of non–unique alignments are allowed. For calculation of confidence and species clusterings, a support score has to be defined to quantify the quality of an individual read alignment. Here, even simple alignment scores may be used, although the resolution of any approach based only on the individual read alignments is necessarily much lower than a more sophisticated approach taking also into account alignments of other reads as used by ContextMap. Thus, the methods proposed in this article will also be helpful for researchers preferring to keep to their already established pipelines and only post–process their results.

## Supporting Information

Figure S1 **Outline of the ContextMap mapping software.**
(PDF)

Figure S2 **Coverage of all species identified for the mock (left) and miR–155 (right) transfected HeLa cells from the study of Gu** ***et al.*** **, respectively.**
(PDF)

Figure S3 **Phylogenetic tree of the species identified by MEGAN4 for the miR–155 transfected HeLa cells.**
(PDF)

Figure S4 **Average mismatch (mm) distributions across all tumor and normal tissue samples in the colorectal carcinoma data set for the three** ***Pseudomonas*** **strains.**
(PDF)

Figure S5 **Phylogenetic tree of the species identified by MEGAN4 on the colorectal carcinoma samples for patient 1 after aligning with megablast against viral and microbial genomes and the human microbiome.**
(PDF)

Figure S6
**Average mismatch (mm) distributions for the microbe and virus hits identified by ContextMap on the microbial community data set.** Results are shown for species with coverage 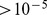 and at least 20 reads.(PDF)

Figure S7 **Phylogenetic tree of the species identified by MEGAN4 for the** ***in–vitro*** **simulated microbial community.**
(PDF)

Table S1 **Microbial and virus species with at least 1000 mapped reads in the mock transfected HeLa cells.**
(PDF)

Table S2 **Microbial and virus species with at least 1000 mapped reads in the miR–155 transfected HeLa cells.**
(PDF)

Table S3 **Species identified by ContextMap in RNA–seq data of tumor and normal tissue for patient 1 from the colorectal carcinoma data set.**
(PDF)

Table S4 **Runtime and memory requirements of ContextMap and all evaluated tools on all three data sets (sorted according to data set size).**
(PDF)

Table S5
**List of microbe and virus hits identified by ContextMap on the** ***in–vitro*** **simulated microbe community data with a coverage 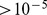 and at least 20 reads.**
(PDF)

Table S6
**List of taxa identified by GASiC with p–value 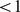.** Species contained in the sample are indicated by an S in the second column.(PDF)

Table S7 **List of taxa identified by GRAMMy with a relative abundance of at least 0.1% (14 out of 63 species identified in total).** Species contained in the sample are indicated by an S in the second column.
(PDF)

Table S8 **Results for MG–RAST on the** ***in–vitro*** **simulated microbial community.** MG–RAST estimates abundance of individual species based on a protein similarity search between predicted proteins and a reference database.
(PDF)

Table S9 **Results for MetaPhyler on the** ***in–vitro*** **simulated microbial community.** MetaPhyler performs taxonomic classification based on phylogenetic marker genes.
(PDF)

Table S10 **Results for SOrt–ITEMS on the** ***in–vitro*** **simulated microbial community.** SOrt–ITEMS assigns reads to a taxon based on significant BLAST hits and performs read assignment at the genus level or higher.
(PDF)

Table S11 **Results for MARTA, an approach for performing taxonomic classification for BLAST hits, on the** ***in–vitro*** **simulated microbial community.**
(PDF)

Table S12 **Results for MLTreeMap on the** ***in–vitro*** **simulated microbial community.** All results with a placement weight of at least 0.05% are shown. Numbers in parenthesis indicate the taxon identifier of the corresponding species. LCA is short for lowest common ancestor.
(PDF)

Table S13 **Results for PhyloPhytiaS, a composition–based approach for species identification, on the** ***in–vitro*** **simulated microbial community.** PhyloPhytiaS performs classification only at the species– not the strain–level.
(PDF)

Table S14 **Results for ClaMS, a composition–based approach, on the** ***in–vitro*** **simulated microbial community.** ClaMS models each sequence as a walk in a de Bruijn graph with underlying Markov chain properties. For each read to be binned, a signature is calculated and compared to a training set of signatures from genome sequence.
(PDF)

Table S15 **Results for Phymm, a composition–based approach, and PhymmBL, a hybrid approach combining Phymm and BLAST results, on the** ***in–vitro*** **simulated microbial community.**
(PDF)
