# Supplementary material for: Mining RNA–Seq Data for Infections and Contaminations
Source: PLoS One. 2013 Sep 3;8(9):e73071. doi: 10.1371/journal.pone.0073071 (PMC3760913; doi:10.1371/journal.pone.0073071)

Figure S1

Outline of the ContextMap mapping software. (1) Within each context, ambiguous mappings are identified for each read with at most a maximum number of mismatches, including both full and spliced alignments. These ambiguous mappings may point to different contexts or may suggest different positions in the same context. (2) The best mapping within each context is identified for each read depending on the support by other reads. (3) Among the mappings to different contexts, the optimal one is chosen resulting in one unique mapping for the read.

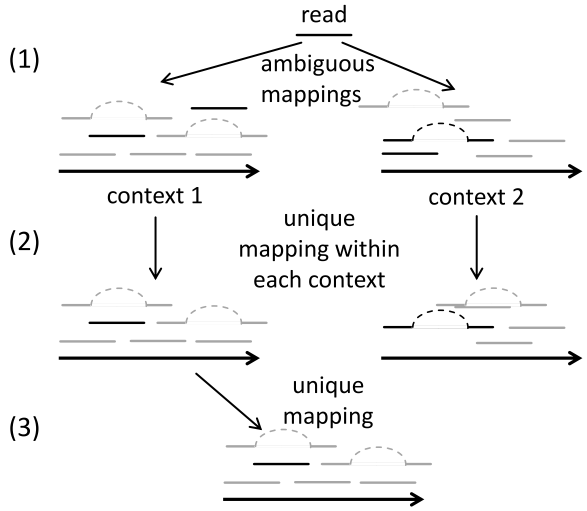

Supplement: Figure S1 — Outline of the ContextMap mapping software. (PDF) [file pone.0073071.s001.pdf]
