# Supplementary material for: Mining RNA–Seq Data for Infections and Contaminations
Source: PLoS One. 2013 Sep 3;8(9):e73071. doi: 10.1371/journal.pone.0073071 (PMC3760913; doi:10.1371/journal.pone.0073071)

Figure S2

Coverage of all species identified for the mock (left) and miR-155 (right) transfected HeLa cells from the study of Gu *et al.*, respectively. The only species identified with a coverage > 0.01 is Human papillomavirus - 18 (indicated in red) with coverage > 0.33.

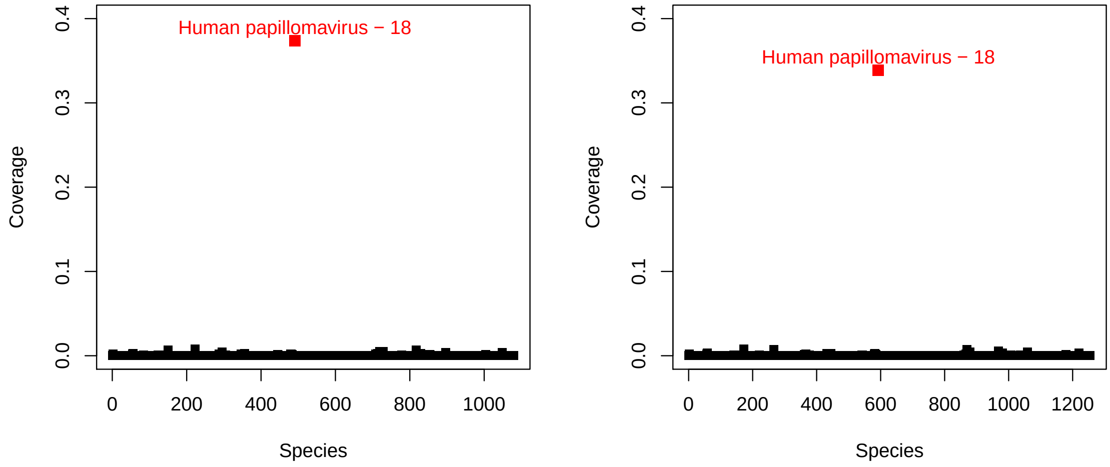

Supplement: Figure S2 — Coverage of all species identified for the mock (left) and miR–155 (right) transfected HeLa cells from the study of Gu et al. , respectively. (PDF) [file pone.0073071.s002.pdf]
