# Supplementary material for: Mining RNA–Seq Data for Infections and Contaminations
Source: PLoS One. 2013 Sep 3;8(9):e73071. doi: 10.1371/journal.pone.0073071 (PMC3760913; doi:10.1371/journal.pone.0073071)

Figure S3

Phylogenetic tree of the species identified by MEGAN4 for the miR-155 transfected HeLa cells. Assigned read numbers are annotated next to the species name and node size is proportional to the number of reads assigned to the node. On the left, result are shown for megablast runs only against microbial and viral genomes, on the right results including also human rRNA and mitochondrial genome sequences. HPV-18 is indicated in red. Blue and green indicates reads mapped to *Rickettsia rickettsii str. Hino* and *Choristoneura occidentalis granulovirus*, respectively, if human sequences are not also used for mapping. If they are used, these reads are assigned to the node "cellular organism" and the root, respectively.

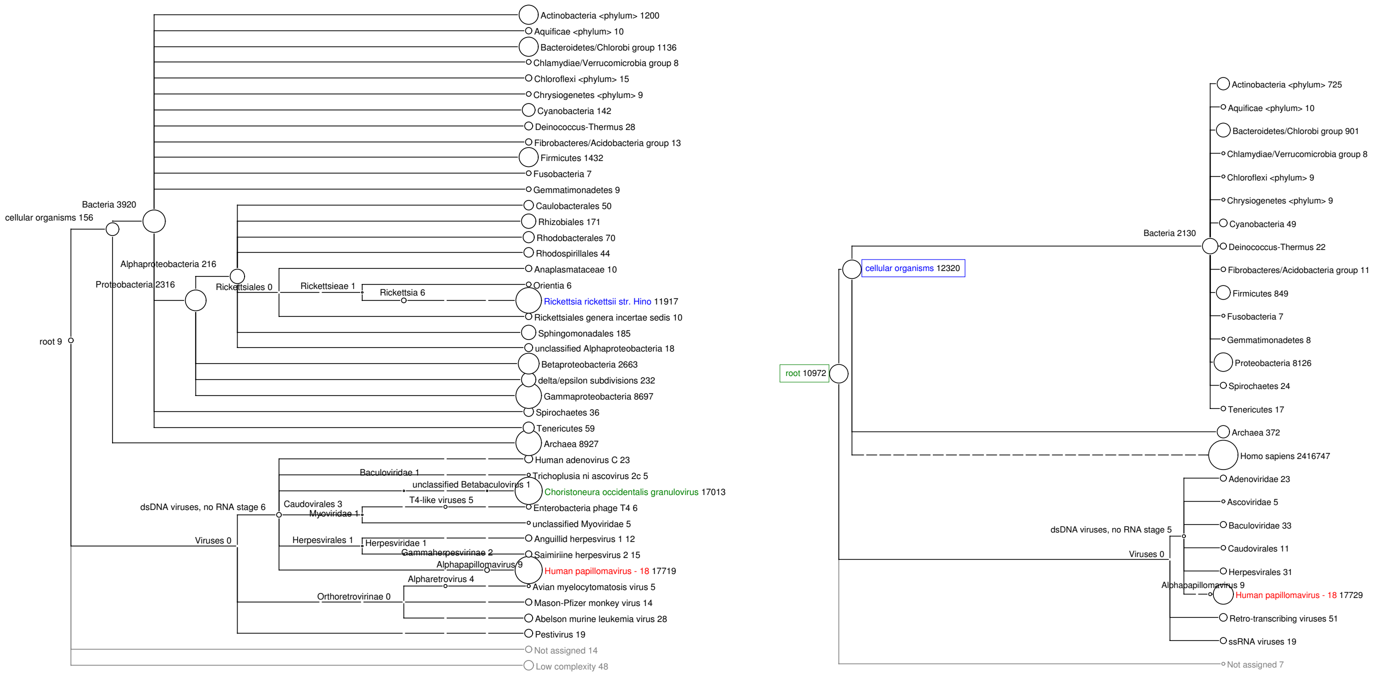

Supplement: Figure S3 — Phylogenetic tree of the species identified by MEGAN4 for the miR–155 transfected HeLa cells. (PDF) [file pone.0073071.s003.pdf]
