# Supplementary material for: Mining RNA–Seq Data for Infections and Contaminations
Source: PLoS One. 2013 Sep 3;8(9):e73071. doi: 10.1371/journal.pone.0073071 (PMC3760913; doi:10.1371/journal.pone.0073071)

Figure S4

Average mismatch (mm) distributions across all tumor and normal tissue samples in the colorectal carcinoma data set for the three *Pseudomonas* strains. Distributions are compared against the average mismatch distribution for the human reference genome. Numbers in parentheses indicate the divergence ($\sqrt{D_{JS}}$) of the mismatch distribution from the reference genome.

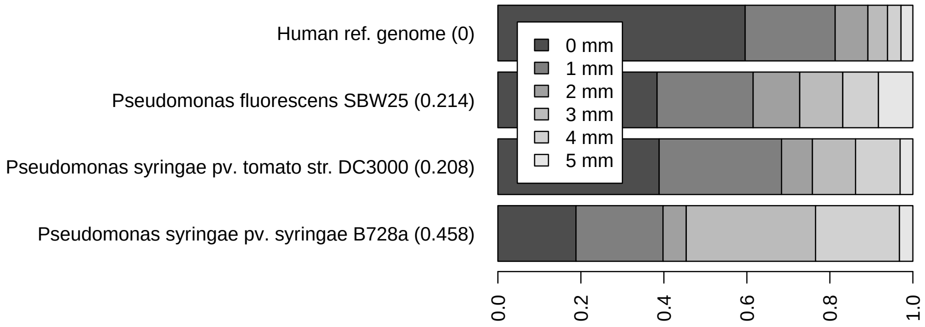

Supplement: Figure S4 — Average mismatch (mm) distributions across all tumor and normal tissue samples in the colorectal carcinoma data set for the three Pseudomonas strains. (PDF) [file pone.0073071.s004.pdf]
