# Supplementary material for: Mining RNA–Seq Data for Infections and Contaminations
Source: PLoS One. 2013 Sep 3;8(9):e73071. doi: 10.1371/journal.pone.0073071 (PMC3760913; doi:10.1371/journal.pone.0073071)

Figure S5

Phylogenetic tree of the species identified by MEGAN4 on the colorectal carcinoma samples for patient 1 after aligning with megablast against viral and microbial genomes and the human microbiome. Only reads were used that were not mapped to human sequences by ContextMap. Assigned read numbers are annotated next to the species name and node size is proportional to the number of reads assigned to the node.

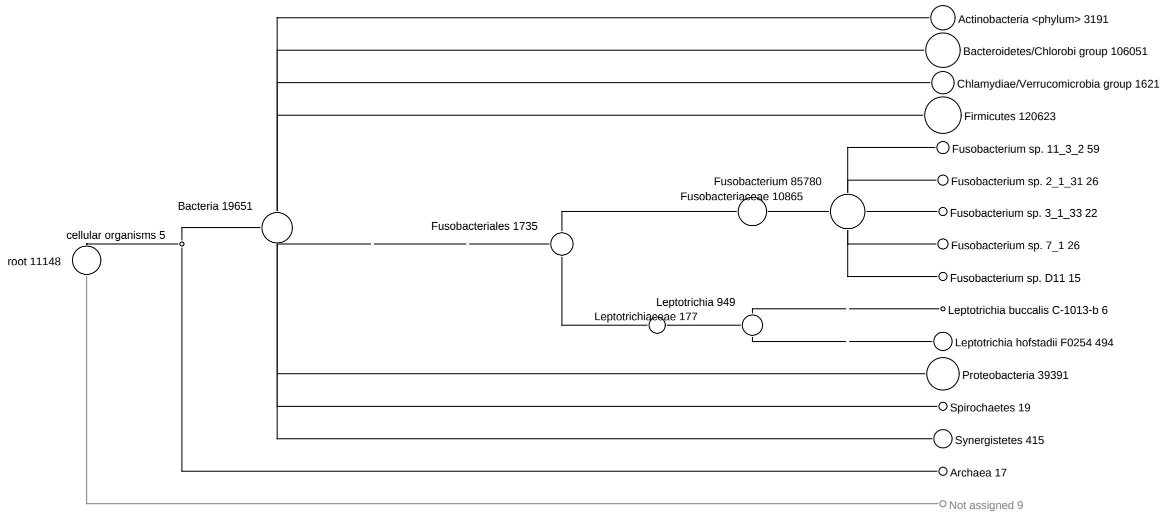

Supplement: Figure S5 — Phylogenetic tree of the species identified by MEGAN4 on the colorectal carcinoma samples for patient 1 after aligning with megablast against viral and microbial genomes and the human microbiome. (PDF) [file pone.0073071.s005.pdf]
