# Supplementary material for: Mining RNA–Seq Data for Infections and Contaminations
Source: PLoS One. 2013 Sep 3;8(9):e73071. doi: 10.1371/journal.pone.0073071 (PMC3760913; doi:10.1371/journal.pone.0073071)

Figure S6

Average mismatch (mm) distributions for the microbe and virus hits identified by ContextMap on the microbial community data set. Results are shown for species with coverage $> 10^{-5}$ and at least 20 reads. Numbers in parentheses indicate the number of reads mapped to the species and the divergence ($\sqrt{D_{JS}}$) of the mismatch distribution from the reference genome, in this case *Myxococcus xanthus DK 1622*.

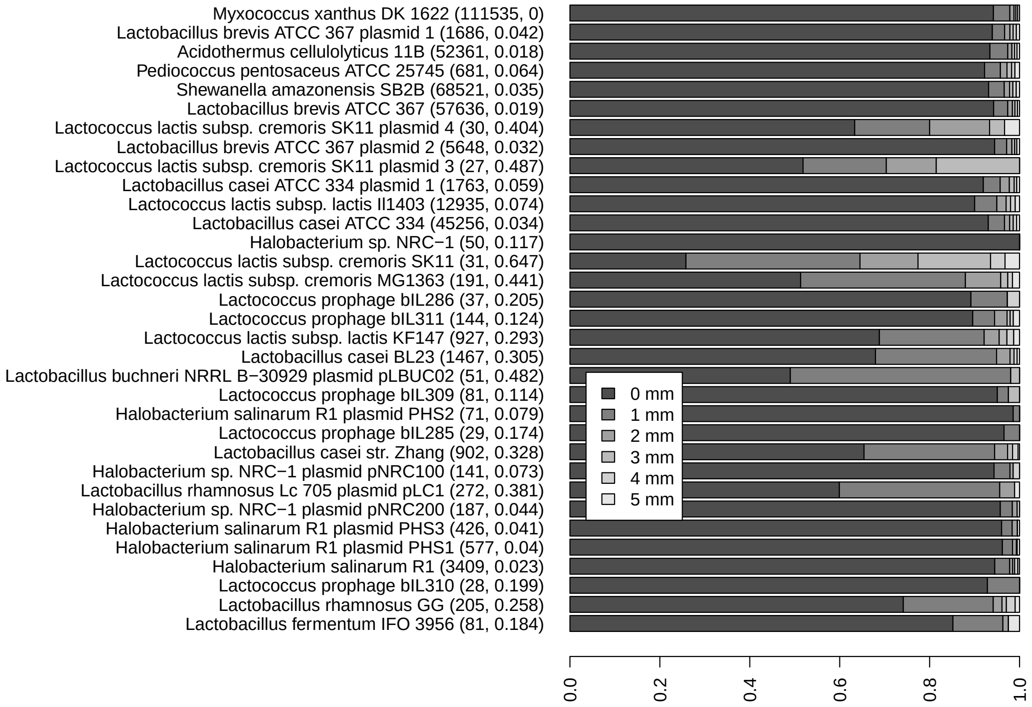

Supplement: Figure S6 — Average mismatch (mm) distributions for the microbe and virus hits identified by ContextMap on the microbial community data set. Results are shown for species with coverage and at least 20 reads. (PDF) [file pone.0073071.s006.pdf]
