# Supplementary material for: Mining RNA–Seq Data for Infections and Contaminations
Source: PLoS One. 2013 Sep 3;8(9):e73071. doi: 10.1371/journal.pone.0073071 (PMC3760913; doi:10.1371/journal.pone.0073071)

Figure S7

Phylogenetic tree of the species identified by MEGAN4 for the *in-vitro* simulated microbial community. Assigned read numbers are annotated next to the species name and node size is proportional to the number of reads assigned to the node. Species contained in the sample are colored in red.

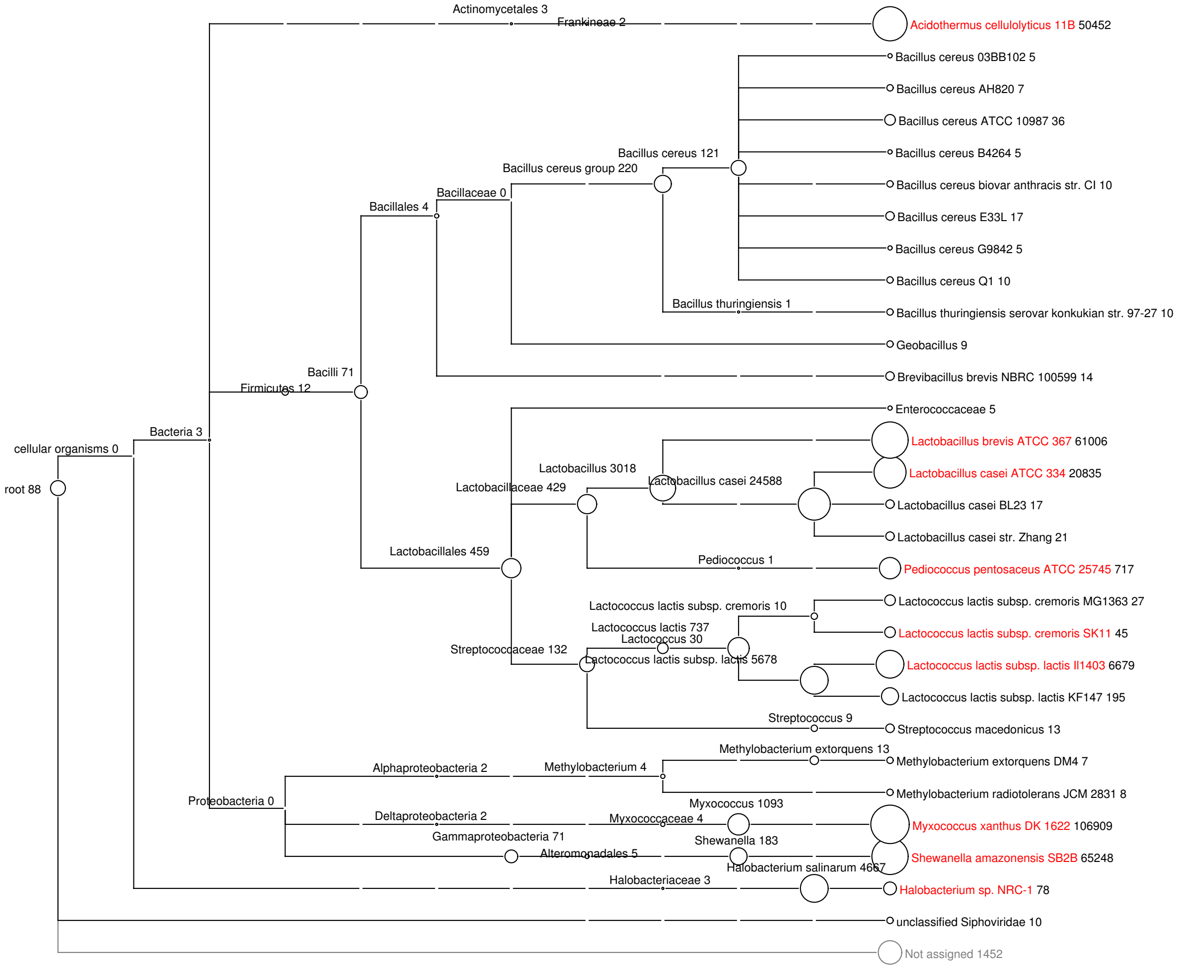

Supplement: Figure S7 — Phylogenetic tree of the species identified by MEGAN4 for the in–vitro simulated microbial community. (PDF) [file pone.0073071.s007.pdf]
