# Supplementary material for: Mining RNA–Seq Data for Infections and Contaminations
Source: PLoS One. 2013 Sep 3;8(9):e73071. doi: 10.1371/journal.pone.0073071 (PMC3760913; doi:10.1371/journal.pone.0073071)
Supplement: Table S1 — Microbial and virus species with at least 1000 mapped reads in the mock transfected HeLa cells. (PDF) [file pone.0073071.s008.pdf]

Table S1

Microbial and virus species with at least 1000 mapped reads in the mock transfected HeLa cells.

species	# reads	coverage	confidence	$\sqrt{D_{JS}}$
Human papillomavirus - 18	22105	3.7e-01	1.000	0.048
Hepatitis C virus genotype 6	1278	2.3e-03	0.558	0.351
Encephalomyocarditis virus	3105	2.4e-03	0.239	0.382
Thermoanaerobacter wiegelii Rt8.B1 chromosome	28366	5.6e-05	0.087	0.460
