# Supplementary material for: Mining RNA–Seq Data for Infections and Contaminations
Source: PLoS One. 2013 Sep 3;8(9):e73071. doi: 10.1371/journal.pone.0073071 (PMC3760913; doi:10.1371/journal.pone.0073071)
Supplement: Table S2 — Microbial and virus species with at least 1000 mapped reads in the miR–155 transfected HeLa cells. (PDF) [file pone.0073071.s009.pdf]

Table S2

Microbial and virus species with at least 1000 mapped reads in the miR-155 transfected HeLa cells.

species	# reads	coverage	confidence	$\sqrt{D_{JS}}$
Human papillomavirus - 18	18491	3.4e-01	1.000	0.045
Choristoneura occidentalis granulovirus	1144	2.4e-04	0.998	0.665
Encephalomyocarditis virus	4130	2.7e-03	0.258	0.418
Acinetobacter sp. ADP1 chromosome	1070	1.4e-04	0.070	0.077
Caviid herpesvirus 2	2215	2.1e-04	0.069	0.463
Thermoanaerobacter wiegelii Rt8.B1 chromosome	24505	5.1e-05	0.063	0.508
Acinetobacter calcoaceticus PHEA-2 chromosome	1480	1.9e-04	0.063	0.120
Acinetobacter baumannii ATCC 17978	1498	1.7e-04	0.020	0.121
