# Supplementary material for: Mining RNA–Seq Data for Infections and Contaminations
Source: PLoS One. 2013 Sep 3;8(9):e73071. doi: 10.1371/journal.pone.0073071 (PMC3760913; doi:10.1371/journal.pone.0073071)
Supplement: Table S3 — Species identified by ContextMap in RNA–seq data of tumor and normal tissue for patient 1 from the colorectal carcinoma data set. (PDF) [file pone.0073071.s010.pdf]

Table S3

Species identified by ContextMap in RNA-seq data of tumor and normal tissue for patient 1 from the colorectal carcinoma data set. This table shows results for all species with at least 1000 mapped reads in at least one sample and $\sqrt{D_{JS}} \leq 0.15$ in the tumor tissue. The second column indicates the database from which the genome sequence was obtained: RS=RefSeq and HMP=the Human Microbiome Project. The last column indicates the enrichment of the particular species in the tumor sample compared to the normal tissue. Only hits with an enrichment ≥ 1 are shown. For calculating enrichment, read numbers were first divided by the number of reads mapped to any species in the corresponding sample. Furthermore, a pseudocount of 5 was used for each sample to address the problem of 0 reads in one sample. This means that enrichment is calculated as

$$\frac{(\#\text{ species reads in tumor} + 5) \cdot (\#\text{ mapped reads for normal tissue})}{(\#\text{ mapped reads for tumor}) \cdot (\#\text{ species reads in normal tissue} + 5)}.$$

species	DB	#reads tumor	$\sqrt{D_{JS}}$ tumor	#reads normal	$\sqrt{D_{JS}}$ normal	enrichment
Escherichia coli 83972	HMP	6707	0.1164	0	NA	1526.07
Escherichia coli MS 200-1	HMP	3844	0.1103	3	0.4135	546.95
Escherichia coli MS 187-1	HMP	1857	0.0835	0	NA	423.35
Selenomonas sputigena ATCC 35185	RS	13586	0.1258	39	0.2827	351.15
ADGF01000000	HMP	1085	0.0347	0	NA	247.83
Fusobacterium sp. 11_3_2	HMP	24678	0.1053	126	0.1255	214.20
Fusobacterium sp. 3_1_33	HMP	50588	0.0730	307	0.1343	184.34
ACAC01000000	HMP	3098	0.1023	25	0.1169	117.59
Bacteroides sp. 2_1	HMP	8436	0.1461	128	0.4059	72.15
ACID01000000	HMP	1169	0.0841	17	0.3428	60.67
Fusobacterium sp. D11	HMP	11375	0.1422	222	0.1167	56.99
Escherichia coli MS 45-1	HMP	1777	0.0506	38	0.0469	47.11
Escherichia coli MS 21-1	HMP	2028	0.1151	54	0.1480	39.17
Granulicatella adiacens ATCC 49175	HMP	3475	0.1031	120	0.0656	31.65
Bacteroides fragilis YCH46	RS	24448	0.1415	927	0.1620	29.83
Bacteroides fragilis NCTC 9343	RS	3605	0.1132	172	0.1178	23.19
Bilophila sp. 4_1_30	HMP	2360	0.1065	123	0.0689	21.00
Bilophila wadsworthia 3_1_6	HMP	1753	0.1255	94	0.2020	20.19
Bacteroides fragilis 638R	RS	7508	0.1325	497	0.1965	17.01
Gemella morbillorum M424	HMP	2130	0.0935	144	0.0869	16.29
Clostridium asparagiforme DSM 15981	HMP	6062	0.0949	424	0.4643	16.08
Solobacterium moorei F0204	HMP	1015	0.1098	88	0.0678	12.47
ACAA01000000	HMP	5239	0.0889	643	0.1148	9.20
Peptostreptococcus stomatis DSM 17678	HMP	1877	0.1233	423	0.0790	5.00
Collinsella aerofaciens ATCC 25986	HMP	1008	0.1308	352	0.1924	3.23
Desulfovibrio piger ATCC 29098	HMP	1253	0.1457	936	0.2271	1.52
