# Supplementary material for: Mining RNA–Seq Data for Infections and Contaminations
Source: PLoS One. 2013 Sep 3;8(9):e73071. doi: 10.1371/journal.pone.0073071 (PMC3760913; doi:10.1371/journal.pone.0073071)
Supplement: Table S4 — Runtime and memory requirements of ContextMap and all evaluated tools on all three data sets (sorted according to data set size). (PDF) [file pone.0073071.s011.pdf]

Table S4

Runtime and memory requirements of ContextMap and all evaluated tools on all three data sets (sorted according to data set size). The third column indicates the reference set. Here, 'genus' indicates that only genomes from the same genus as the microbes in the simulated microbial community were used.

Both the maximum virtual memory and the resident set size (portion of a process's memory held in RAM) are shown. For programs implemented in Java (ContextMap, MARTA, ClaMS) the latter is more informative, as Java will allocate the amount of memory provided by the -Xmx option regardless of whether it needs it or not. For runtime both real and user CPU time were determined using the unix program 'time' and rounded to minutes.

The GRAMMy and GASiC runs on all species were aborted after 48 hours without results. Thus, only the results for a mapping against the 'genus' set is shown. Runtime and memory to obtain the BLAST input for GRAMMy are not included in its runtime and memory. MG-RAST and PhyloPhytiaS are provided as web servers and thus could not be evaluated.

For BLAST-based approaches that perform analysis individually for each read (BLAST, Phymm/PhymmBL, MARTA, and SOrt-ITEMS), the read set was split into five subsets to perform some parallelization and approaches were run separately on each read set. CPU times for the five runs were added up and the average memory of any of the runs is shown in the table. Please note that this a lower bound on the maximum memory required as all reads combined may require more memory.

Program	# Reads	Reference Set	Max Virtual memory [MB]	Max Resident Set Size [MB]	Real Time [min]	User CPU Time [min]
Simulated microbial community						
ContextMap	484,629	microbes, viruses, yeast	5295	2489	9	29
GASiC	484,629	genus	3018	2821	298	905
GRAMMy	484,629	genus	588	331	443	443
BLAST (megablast)	484,629	microbes	2568	2440	43	43
MetaPhyler	484,629	microbes	3538	3478	1	2
SOrt-ITEMS	484,629	microbes	2975	2915	1594	677
MARTA	484,629	microbes	6845	4870	2207	13164
MLTreeMap	484,629	microbes	287	224	2659	2636
ClaMS	484,629	microbes	47153	34050	138	275
Phymm/PhymmBL	484,629	microbes	35865	35819	5666	5528
RNA-seq of colorectal carcinoma (Patient 1)						
ContextMap	5,343,842	rDNA, human genome, microbes, human microbiome, viruses	16480	10641	276	1771
BLAST (megablast)	404,234	microbes, human microbiome, viruses	29766	28612	1301	1288
Novoalign	404,234	microbes, human microbiome, viruses	15223	15127	42	38
RNA-seq of HeLa-cells (miR-155 set)						
ContextMap	29,595,334	rRNA, human genome, microbes, viruses	25077	15957	1358	8336
BLAST (megablast)	29,595,334	rRNA, mtDNA, microbes, viruses	2495	2382	497	493
