# Supplementary material for: Mining RNA–Seq Data for Infections and Contaminations
Source: PLoS One. 2013 Sep 3;8(9):e73071. doi: 10.1371/journal.pone.0073071 (PMC3760913; doi:10.1371/journal.pone.0073071)
Supplement: Table S5 — List of microbe and virus hits identified by ContextMap on the in–vitro simulated microbe community data with a coverage and at least 20 reads. (PDF) [file pone.0073071.s012.pdf]

Table S5

List of microbe and virus hits identified by ContextMap on the *in-vitro* simulated microbe community data with a coverage $> 10^{-5}$ and at least 20 reads. Entries are sorted according to ContextMap confidence. The type of the hit is indicated in the following way: S = the species is contained in the sample; R = a close relation is contained in the sample; R? = a more distant relation is contained in the sample; P = a prophage of a species in the sample. For both ContextMap and BLAST, the number of reads mapped to the species and the confidence are provided. In case of BLAST, the number of mapped reads includes also reads that can be mapped equally well to any other species, thus including multiple mappings. BLAST confidence is defined as the fraction of reads that can be mapped uniquely to this species using BLAST.

Species	Type	Coverage	ContextMap #reads	ContextMap confid.	$\sqrt{D_{JS}}$	BLAST #reads	BLAST conf.
Lactobacillus brevis ATCC 367 plasmid 1	S	1.1e-01	1686	1.000	0.042	1605	1.00000
Acidothermus cellulolyticus 11B	S	2.0e-02	52361	1.000	0.018	50458	0.99988
Pediococcus pentosaceus ATCC 25745	S	3.4e-04	681	0.999	0.064	843	0.85053
Shewanella amazonensis SB2B	S	1.5e-02	68521	0.997	0.035	65504	0.99609
Lactobacillus brevis ATCC 367	S	2.3e-02	57636	0.992	0.019	56240	0.98131
Myxococcus xanthus DK 1622	S	1.1e-02	111535	0.985	0.000	108008	0.98982
Lactococcus lactis subsp. cremoris SK11 plasmid 4	S	6.1e-04	30	0.956	0.404	101	0.24752
Lactobacillus brevis ATCC 367 plasmid 2	S	1.4e-01	5648	0.768	0.032	5449	0.76766
Lactococcus lactis subsp. cremoris SK11 plasmid 3	S	3.1e-04	27	0.587	0.487	117	0.11111
Lactobacillus casei ATCC 334 plasmid 1	S	5.4e-02	1763	0.567	0.059	1917	0.54199
Lactococcus lactis subsp. lactis Il1403	S	5.1e-03	12935	0.435	0.074	13305	0.45133
Lactobacillus casei ATCC 334	S	1.4e-02	45256	0.393	0.034	46082	0.42958
Halobacterium sp. NRC-1	S	2.2e-05	50	0.370	0.117	3456	0.00376
Lactococcus lactis subsp. cremoris SK11	S	1.2e-05	31	0.344	0.647	658	0.00912
Lactococcus lactis subsp. cremoris MG1363	R	6.8e-05	191	0.262	0.441	871	0.03100
Lactococcus prophage bIL286	P	8.6e-04	37	0.195	0.205	74	0.00000
Lactococcus prophage bIL311	P	8.8e-03	144	0.182	0.124	166	0.00000
Lactococcus lactis subsp. lactis KF147	R	3.3e-04	927	0.171	0.293	6537	0.02937
Lactobacillus casei BL23	R	4.3e-04	1467	0.153	0.305	21019	0.00081
Lactobacillus buchneri NRRL B-30929 plasmid pLBUC02	R?	2.3e-03	51	0.148	0.482	640	0.00000
Lactococcus prophage bIL309	P	2.0e-03	81	0.138	0.114	205	0.00976
Halobacterium salinarum R1 plasmid PHS2	R	3.5e-04	71	0.121	0.079	540	0.00000
Lactococcus prophage bIL285	P	8.2e-04	29	0.116	0.174	176	0.00000
Lactobacillus casei str. Zhang	R	2.8e-04	902	0.110	0.328	18885	0.00111
Halobacterium sp. NRC-1 plasmid pNRC100	S	7.1e-04	141	0.109	0.073	959	0.00104
Lactobacillus rhamnosus Lc 705 plasmid pLC1	R?	3.2e-03	272	0.105	0.381	884	0.00000
Halobacterium sp. NRC-1 plasmid pNRC200	S	4.9e-04	187	0.093	0.044	1269	0.00079
Halobacterium salinarum R1 plasmid PHS3	R	1.4e-03	426	0.090	0.041	509	0.00000
Halobacterium salinarum R1 plasmid PHS1	R	3.6e-03	577	0.084	0.040	823	0.00000
Halobacterium salinarum R1	R	1.6e-03	3409	0.070	0.023	3414	0.00029
Lactococcus prophage bIL310	P	1.8e-03	28	0.041	0.199	101	0.00000
Lactobacillus rhamnosus GG	R?	5.0e-05	205	0.034	0.258	1220	0.00000
Lactobacillus fermentum IFO 3956	R?	2.7e-05	81	0.022	0.184	692	0.00000
