# Supplementary material for: Mining RNA–Seq Data for Infections and Contaminations
Source: PLoS One. 2013 Sep 3;8(9):e73071. doi: 10.1371/journal.pone.0073071 (PMC3760913; doi:10.1371/journal.pone.0073071)
Supplement: Table S6 — List of taxa identified by GASiC with p–value . Species contained in the sample are indicated by an S in the second column. (PDF) [file pone.0073071.s013.pdf]

Table S6

List of taxa identified by GASiC with p-value < 1. Species contained in the sample are indicated by an S in the second column. Please note that GASiC performs mapping independently for each species. Thus, reads can be mapped to more than one species. An additional 113 species identified by GASiC with a p-value of 1 are not shown.

Species	Type	# reads	P-value
Lactobacillus casei BL23		41484	0.89
Lactobacillus casei str. Zhang		38760	0.97
Lactococcus lactis subsp. lactis Il1403	S	14438	0.00
Shewanella amazonensis SB2B	S	68526	0.00
Lactobacillus brevis ATCC 367	S	58571	0.00
Lactobacillus brevis ATCC 367 plasmid 2	S	5700	0.00
Acidothermus cellulolyticus 11B	S	52376	0.00
Myxococcus xanthus DK 1622	S	111547	0.00
Lactobacillus casei ATCC 334	S	48607	0.00
