# Supplementary material for: Mining RNA–Seq Data for Infections and Contaminations
Source: PLoS One. 2013 Sep 3;8(9):e73071. doi: 10.1371/journal.pone.0073071 (PMC3760913; doi:10.1371/journal.pone.0073071)
Supplement: Table S7 — List of taxa identified by GRAMMy with a relative abundance of at least 0.1% (14 out of 63 species identified in total). Species contained in the sample are indicated by an S in the second column. (PDF) [file pone.0073071.s014.pdf]

Table S7

List of taxa identified by GRAMMy with a relative abundance of at least 0.1% (14 out of 63 species identified in total). Species contained in the sample are indicated by an S in the second column. GRAMMy only estimates relative abundances of species in the sample from alignments (in this case BLAST alignments), but performs no resolution of non-unique mappings.

Species	Type	Abundance
Lactobacillus brevis ATCC 367	S	0.27110
Acidothermus cellulolyticus 11B	S	0.20900
Lactobacillus casei ATCC 334	S	0.16500
Shewanella amazonensis SB2B	S	0.15400
Myxococcus xanthus DK 1622	S	0.11970
Lactococcus lactis subsp. lactis Il1403	S	0.05579
Halobacterium sp. NRC-1	S	0.01847
Pediococcus pentosaceus ATCC 25745	S	0.00400
Lactococcus lactis subsp. lactis KF147		0.00169
Lactococcus lactis subsp. cremoris SK11	S	0.00036
Lactobacillus casei str. Zhang		0.00028
Lactobacillus casei BL23		0.00023
Halobacterium salinarum R1		0.00022
Lactococcus lactis subsp. cremoris MG1363		0.00016
