# Supplementary material for: Mining RNA–Seq Data for Infections and Contaminations
Source: PLoS One. 2013 Sep 3;8(9):e73071. doi: 10.1371/journal.pone.0073071 (PMC3760913; doi:10.1371/journal.pone.0073071)
Supplement: Table S8 — Results for MG–RAST on the in–vitro simulated microbial community. MG–RAST estimates abundance of individual species based on a protein similarity search between predicted proteins and a reference database. (PDF) [file pone.0073071.s015.pdf]

Table S8

This table shows the evaluation results for MG-RAST on the *in-vitro* simulated microbial community. MG-RAST estimates abundance of individual species based on a protein similarity search between predicted proteins and a reference database. Here, we used the following cutoffs: maximum E-value = 1e-5, minimum identity = 60%, minimum alignment length = 15 amino acids and minimum abundance=20. As MG-RAST only identifies species but not individual strains, it cannot distinguish the *cremoris SK11* subspecies. Furthermore, several species not contained in the sample were found to be more abundant than *Pediococcus pentosaceus* and *Halobacterium sp.*, which are contained in the sample. The latter probably represents *Halobacterium sp. NRC-1*, whereas *Halobacterium salinarum*, which was also found, probably represents the *R1* strain.

species	abundance	avg. E-value	avg. % ident	avg. alignment length	# hits
Myxococcus xanthus	19161	-7.07	98.75	27.24	9355
Shewanella amazonensis	6438	-7.16	99.12	27.27	3195
Lactobacillus brevis	5416	-7.34	97.35	27.73	2481
Lactobacillus casei	4837	-6.44	98.93	26.08	2899
Acidothermus cellulolyticus	4311	-6.24	99.15	25.76	2367
Lactococcus lactis	2014	-6.26	97.75	26.23	1828
Lactobacillus paracasei	1567	-6.3	98.91	25.94	1129
Halobacterium salinarum	590	-7.26	98.99	27.43	590
unassigned	566	-6.66	92.85	27.72	566
Stigmatella aurantiaca	223	-5.54	87.66	26.58	223
Shewanella baltica	181	-6.25	97.78	25.74	181
Saccharomyces cerevisiae	175	-7.49	98.12	27.51	175
Pediococcus pentosaceus	144	-6.18	98.16	25.4	144
Shewanella sp.	142	-5.37	96.2	25.02	142
Brevibacillus brevis	132	-5.45	85.71	27.55	132
Shewanella putrefaciens	112	-5.85	95.03	25.81	112
Shewanella oneidensis	100	-5.77	93.95	26.01	100
Lactobacillus plantarum	94	-5.39	93.05	25.71	94
Bacillus thuringiensis	72	-6.04	97.58	25.19	72
Halobacterium sp.	70	-6.98	99.02	26.87	68
Shewanella violacea	46	-5.71	87.91	27.46	46
Shewanella loihica	44	-5.81	96.85	25.45	44
Shewanella sp. W3-18-1	42	-5.37	96.69	24.87	42
Shewanella denitrificans	41	-5.84	94.37	25.65	41
Shewanella sp. MR-7	41	-5.16	98.14	24.37	41
Shewanella sp. MR-4	39	-5.44	96.77	24.93	39
Shewanella pealeana	36	-5.23	97.08	24.24	36
Shewanella sp. ANA-3	36	-5.75	98	25.4	36
Shewanella frigidimarina	33	-5.12	91.54	25.57	33
Enterococcus faecalis	31	-6.19	93.8	27.54	31
Shewanella sediminis	27	-5.67	96.81	24.96	27
Bacillus cereus	25	-6.03	95.82	26.17	25
Vibrio cholerae	21	-6.68	97.06	27.24	21
