# Supplementary material for: Mining RNA–Seq Data for Infections and Contaminations
Source: PLoS One. 2013 Sep 3;8(9):e73071. doi: 10.1371/journal.pone.0073071 (PMC3760913; doi:10.1371/journal.pone.0073071)
Supplement: Table S9 — Results for MetaPhyler on the in–vitro simulated microbial community. MetaPhyler performs taxonomic classification based on phylogenetic marker genes. (PDF) [file pone.0073071.s016.pdf]

Table S9

This table shows the results for MetaPhyler on the *in-vitro* simulated microbial community. MetaPhyler performs taxonomic classification based on phylogenetic marker genes. As a consequence, the number of reads assigned is relatively small, as few originate from the marker genes. Furthermore, MetaPhyler only performs classification of the genus and not species or strains. Thus, performance in distinguishing the *Lactococcus* and *Halobacterium* species/strains cannot be evaluated. The genera contained in the sample are correctly identified with the exception of *Bacillus* (which is found to be more frequent than *Pediococcus*) and *Sphingomonas*.

genus	% abundance	depth of coverage	number of reads	similarity with reference
Lactobacillus	45.77	3.74	666	99.33
Acidothermus	17.81	1.45	296	99.58
Shewanella	17.1	1.39	289	99.4
Lactococcus	9.25	0.75	143	99.58
Myxococcus	7.43	0.6	144	99.66
Halobacterium	1.16	0.09	18	99.77
Myxococcales{order}	0.37	0.03	7	92.14
Bacillus	0.33	0.02	4	98.75
Pediococcus	0.24	0.01	3	100
Actinomycetales{order}	0.2	0.01	4	91.25
Firmicutes{phylum}	0.13	0.01	2	90
Gammaproteobacteria{class}	0.06	0	1	90
Sphingomonas	0.05	0	1	96
Halobacteriaceae{family}	0.04	0	1	93
