# Supplementary material for: Mining RNA–Seq Data for Infections and Contaminations
Source: PLoS One. 2013 Sep 3;8(9):e73071. doi: 10.1371/journal.pone.0073071 (PMC3760913; doi:10.1371/journal.pone.0073071)
Supplement: Table S10 — Results for SOrt–ITEMS on the in–vitro simulated microbial community. SOrt–ITEMS assigns reads to a taxon based on significant BLAST hits and performs read assignment at the genus level or higher. (PDF) [file pone.0073071.s017.pdf]

Table S10

This table shows the results for SOrt-ITEMS on the *in-vitro* simulated microbial community. SOrt-ITEMS assigns reads to a taxon based on significant BLAST hits and performs read assignment at the genus level or higher. All hits with at least 100 assigned reads are shown. The top-ranked hits indeed correspond to genera contained in the sample. However, *Pediococcus* is ranked very low, below other taxa not contained in the sample.

species	read count
Lactobacillus	115169
Myxococcus	89568
Shewanella	66810
Acidothermus	43734
Lactococcus	14820
Myxococcales	10574
Lactobacillaceae	4225
Halobacterium	3699
cellular organisms	3654
Lactobacillales	3312
Gammaproteobacteria	2906
Myxococcaceae	2692
Bacteria	2638
Cystobacterineae	2343
Actinomycetales	2090
Bacillus	1544
Proteobacteria	1219
Shewanellaceae	1196
Firmicutes	1096
Saccharomyces	1076
Streptococcaceae	1022
Paenibacillaceae	878
Pediococcus	841
root	791
Alteromonadales	754
Halobacteriaceae	655
Bacilli	498
Bacillaceae	329
Acidothermaceae	320
Streptococcus	315
Bacillales	272
Chondromyces	262
Enterobacteriaceae	258
Brevibacillus	252
Actinobacteria	171
Enterococcus	167
Vibrio	145
Polyangiaceae	140
Frankineae	137
Streptomyces	103
