# Supplementary material for: Mining RNA–Seq Data for Infections and Contaminations
Source: PLoS One. 2013 Sep 3;8(9):e73071. doi: 10.1371/journal.pone.0073071 (PMC3760913; doi:10.1371/journal.pone.0073071)
Supplement: Table S11 — Results for MARTA, an approach for performing taxonomic classification for BLAST hits, on the in–vitro simulated microbial community. (PDF) [file pone.0073071.s018.pdf]

Table S11

This table shows the results for MARTA, an approach for performing taxonomic classification for BLAST hits, on the *in-vitro* simulated microbial community. All identified species with >10 assigned reads are shown. MARTA performs classification only at the species- not strain-level, thus performance in distinguishing *Halobacterium sp. NRC-1* and *Lactococcus lactis subsp. cremoris SK11* cannot be evaluated. However, all 8 species contained in the sample are ranked higher than all other species in terms of read counts.

species	read count	avg. E-value	avg. score
Myxococcus xanthus	82489	8.3e-36	196
Shewanella amazonensis	52513	3.1e-35	194
Lactobacillus brevis	49061	1.5e-35	195
Acidothermus cellulolyticus	39097	1.1e-35	195
Lactobacillus casei	36942	3.2e-35	194
Lactococcus lactis	11457	6.0e-35	193
Halobacterium salinarum	3622	1.1e-35	196
Pediococcus pentosaceus	630	1.3e-34	192
Bacillus cereus	335	6.7e-34	181
Lactobacillus rhamnosus	215	1.9e-37	192
Pediococcus claussenii	88	4.6e-43	195
Lactobacillus plantarum	63	9.5e-36	194
Lactobacillus buchneri	55	1.8e-33	190
Lactobacillus fermentum	51	1.2e-38	192
Lactobacillus helveticus	48	8.3e-37	189
Myxococcus fulvus	43	2.1e-34	178
Methylobacterium extorquens	24	8.9e-42	187
Lactobacillus delbrueckii	17	1.8e-43	195
Bacillus anthracis	17	1.2e-34	175
Streptococcus thermophilus	16	3.8e-38	192
Bacillus thuringiensis	14	1.4e-36	176
Methylobacterium radiotolerans	12	1.7e-37	186
Brevibacillus brevis	11	1.8e-38	185
