# Supplementary material for: Mining RNA–Seq Data for Infections and Contaminations
Source: PLoS One. 2013 Sep 3;8(9):e73071. doi: 10.1371/journal.pone.0073071 (PMC3760913; doi:10.1371/journal.pone.0073071)
Supplement: Table S12 — Results for MLTreeMap on the in–vitro simulated microbial community. All results with a placement weight of at least 0.05% are shown. Numbers in parenthesis indicate the taxon identifier of the corresponding species. LCA is short for lowest common ancestor. (PDF) [file pone.0073071.s019.pdf]

Table S12

This table shows the results for MLTreeMap on the *in-vitro* simulated microbial community. All results with a placement weight of at least 0.05% are shown. Numbers in parenthesis indicate the taxon identifier of the corresponding species. LCA is short for lowest common ancestor. Although only species names are provided by MLTreeMap, classification is performed at the strain-level as indicated by the taxon identifiers. Here, the highest-ranking hits are enriched for the correct species but often only the correct genus is identified. The following species/strains are missed: *Lactobacillus brevis*, *Lactobacillus casei*, *Lactobacillus casei*, *Lactococcus lactis subsp. cremoris SK11*, and *Shewanella amazonensis*. Furthermore, *Halobacterium sp.* is ranked below *Enterococcus faecalis*, which is not contained in the sample.

placement weight [%]	species
22.1802	Acidothermus cellulolyticus (351607)
12.2243	Myxococcus xanthus (246197)
9.0737	Shewanella oneidensis (211586)
9.0737	Lactococcus lactis 1403 (272623)
9.0107	Lactobacillus plantarum (220668)
6.4902	Pediococcus pentosaceus (278197)
2.8355	LCA of Lactobacillus plantarum (220668) and Pediococcus pentosaceus (278197)
1.8904	Enterococcus faecalis (226185)
1.7013	Halobacterium sp. (64091)
1.5753	LCA of Leuconostoc mesenteroides (203120) and Oenococcus oeni (203123)
1.5123	LCA of Leuconostoc mesenteroides (203120), Oenococcus oeni (203123), Lactobacillus plantarum (220668) and Pediococcus pentosaceus (278197)
1.1972	Streptococcus pneumoniae TIGR4 (170187)
1.0082	LCA of Streptococcus pneumoniae TIGR4 (170187), Leuconostoc mesenteroides (203120), Oenococcus oeni (203123), Lactobacillus plantarum (220668), Enterococcus faecalis (226185), Lactococcus lactis 1403 (272623) and Pediococcus pentosaceus (278197)
0.9452	Leuconostoc mesenteroides (203120)
0.8822	Oenococcus oeni (203123)
0.7561	Pseudoalteromonas haloplanktis (326442)
0.6931	LCA of Wigglesworthia glossinidia (36870), Haemophilus influenzae KW20 (71421), Escherichia coli K12 (83333), Buchnera aphidicola APS (107806), Colwellia psychrerythraea (167879), Shigella flexneri 301 (198214), Blochmannia floridanus (203907), Shewanella oneidensis (211586), Yersinia pestis CO92 (214092), Erwinia carotovora (218491), Salmonella enterica CT18 (220341), Mannheimia succiniciproducens (221988), Photorhabdus luminescens (243265), Vibrio cholerae N16961 (243277), Klebsiella pneumoniae (272620), Pasteurella multocida (272843), Citrobacter koseri (290338), Enterobacter sakazakii (290339), Photobacterium profundum (298386), Pseudoalteromonas haloplanktis (326442), Sodalis glossinidius (343509), Psychromonas ingrahamii (357804), Aeromonas hydrophila (380703), Serratia proteamaculans (399741) and Actinobacillus pleuropneumoniae (416269)
0.6301	Photobacterium profundum (298386)
0.6301	Listeria innocua (272626)
0.5671	Frankia sp. CcI3 (106370)
0.5041	LCA of Streptococcus pneumoniae TIGR4 (170187), Enterococcus faecalis (226185) and Lactococcus lactis 1403 (272623)
0.5041	LCA of Shewanella oneidensis (211586) and Psychromonas ingrahamii (357804)
0.5041	LCA of Escherichia coli K12 (83333), Shigella flexneri 301 (198214), Salmonella enterica CT18 (220341) and Citrobacter koseri (290338)
0.5041	Psychromonas ingrahamii (357804)
0.5041	Geobacillus kaustophilus (235909)
0.5041	Bacillus subtilis (224308)
0.5041	Aeromonas hydrophila (380703)
