# Supplementary material for: Mining RNA–Seq Data for Infections and Contaminations
Source: PLoS One. 2013 Sep 3;8(9):e73071. doi: 10.1371/journal.pone.0073071 (PMC3760913; doi:10.1371/journal.pone.0073071)
Supplement: Table S13 — Results for PhyloPhytiaS, a composition–based approach for species identification, on the in–vitro simulated microbial community. PhyloPhytiaS performs classification only at the species– not the strain–level. (PDF) [file pone.0073071.s020.pdf]

Table S13

This table shows the results for PhyloPhytiaS, a composition-based approach for species identification, on the *in-vitro* simulated microbial community. PhyloPhytiaS performs classification only at the species- not the strain-level. Here, results were obtained using the generic model provided by the webserver and all hits with > 100 reads were retained. With the exception of two species, none of these predicted species are contained in the sample.

species	read count
Desulfovibrio vulgaris	3063
Lactobacillus plantarum	2172
Lactobacillus casei	2170
Bifidobacterium animalis	1972
Bifidobacterium longum	1814
Xylella fastidiosa	1266
Lactococcus lactis	1046
Streptococcus equi	1020
Lactobacillus delbrueckii	740
Streptococcus suis	609
Pseudomonas putida	524
Pseudomonas aeruginosa	514
Vibrio cholerae	510
Prochlorococcus	409
Bacillus subtilis	398
Rhodobacter sphaeroides	363
Streptococcus pyogenes	317
Methanococcus maripaludis	316
Shewanella baltica	226
Clostridium difficile	178
Neisseria meningitidis	167
Mycobacterium tuberculosis	153
Streptococcus thermophilus	134
Haemophilus influenzae	131
Mycobacterium bovis	127
Actinobacillus pleuropneumoniae	127
Vibrio vulnificus	124
Xanthomonas oryzae	121
Streptococcus agalactiae	117
Francisella tularensis	112
Helicobacter pylori	110
Streptococcus pneumoniae	110
