# Supplementary material for: Mining RNA–Seq Data for Infections and Contaminations
Source: PLoS One. 2013 Sep 3;8(9):e73071. doi: 10.1371/journal.pone.0073071 (PMC3760913; doi:10.1371/journal.pone.0073071)
Supplement: Table S14 — Results for ClaMS, a composition–based approach, on the in–vitro simulated microbial community. ClaMS models each sequence as a walk in a de Bruijn graph with underlying Markov chain properties. For each read to be binned, a signature is calculated and compared to a training set of signatures from genome sequence. (PDF) [file pone.0073071.s021.pdf]

Table S14

This table shows the results for ClaMS, a composition-based approach, on the *in-vitro* simulated microbial community. ClaMS models each sequence as a walk in a de Bruijn graph with underlying Markov chain properties. For each read to be binned, a signature is calculated and compared to a training set of signatures from genome sequence. If the normalized distance to the best signature match exceeds a certain threshold, it is assigned to this genome, otherwise the sequence is not binned. Here, we used a distance cutoff of 0.05 as the recommended cutoff of 0.01 resulted in no assigned reads. In the table all all hits with > 3000 reads are shown. As can be seen, none of these are are contained in the sample and only one belongs to a correct genus.

species	read count	average distance
Herpetosiphon aurantiacus DSM 785	30551	0.018
Dichelobacter nodosus VCS1703A	16059	0.019
Acidaminococcus fermentans DSM 20731	11506	0.018
Conexibacter woesei DSM 14684	11443	0.024
Synechococcus sp. RCC307	10286	0.018
Kribbella flavida DSM 17836	10142	0.022
Stenotrophomonas maltophilia JV3	9192	0.021
Stenotrophomonas maltophilia R551-3	9127	0.020
Moraxella catarrhalis RH4	8709	0.020
Cellulomonas fimi ATCC 484	8615	0.028
Leptothrix cholodnii SP-6	8414	0.021
Mycoplasma gallisepticum str. R(low)	8061	0.020
Beutenbergia cavernae DSM 12333	7185	0.025
Spirochaeta thermophila DSM 6192	6698	0.019
Methylibium petroleiphilum PM1	6696	0.018
Myxococcus fulvus HW-1	6468	0.019
Kineococcus radiotolerans SRS30216 plasmid pKRAD02	6358	0.024
Mycoplasma suis str. Illinois	6296	0.022
Treponema brennaborense DSM 12168	6075	0.019
Helicobacter pylori B8 plasmid HPB8p	5926	0.019
Anaeromyxobacter sp. Fw109-5	5803	0.025
Thermus thermophilus HB8	5506	0.026
Anaeromyxobacter dehalogenans 2CP-C	5395	0.027
Phenylobacterium zucineum HLK1	5089	0.022
Eubacterium eligens ATCC 27750 plasmid unnamed	5058	0.020
Nitrosopumilus maritimus SCM1	4950	0.021
Ramlibacter tataouinensis TTB310	4895	0.020
Micromonospora sp. L5	4785	0.022
Sanguibacter keddieii DSM 10542	4046	0.022
Cellvibrio gilvus ATCC 13127	3985	0.024
Blattabacterium sp. (Mastotermes darwiniensis) str. MADAR plasmid pMADAR_001	3714	0.020
Halogeometricum borinquense DSM 11551 plasmid pHBOR05	3646	0.019
Cellulomonas flavigena DSM 20109	3616	0.026
Mycobacterium ulcerans AGY99 plasmid pMUM001	3393	0.017
Brevundimonas subvibrioides ATCC 15264	3359	0.019
Rhodospirillum centenum SW	3295	0.021
Escherichia coli O26:H11 str. 11368 plasmid pO26_2	3292	0.018
Persephonella marina EX-H1	3275	0.017
Nakamurella multipartita DSM 44233	3255	0.019
Candidatus Riesia pediculicola USDA plasmid pPAN	3214	0.022
Staphylococcus epidermidis ATCC 12228 plasmid pSE-12228-03	3135	0.019
Helicobacter bizzozeronii CIII-1	3065	0.016
