# Supplementary material for: Mining RNA–Seq Data for Infections and Contaminations
Source: PLoS One. 2013 Sep 3;8(9):e73071. doi: 10.1371/journal.pone.0073071 (PMC3760913; doi:10.1371/journal.pone.0073071)
Supplement: Table S15 — Results for Phymm, a composition–based approach, and PhymmBL, a hybrid approach combining Phymm and BLAST results, on the in–vitro simulated microbial community. (PDF) [file pone.0073071.s022.pdf]

## Table S15

This table shows the results for Phymm, a composition-based approach, and PhymmBL, a hybrid approach combining Phymm and BLAST results, on the *in-vitro* simulated microbial community. The top 30 hits for either method are listed. In both cases, the correct strains are enriched towards the top of the tables. However, a number of related species or strains are ranked higher than correct hits, in particular higher than *Halobacterium sp. NRC-1*, *Pediococcus pentosaceus*, and *Lactococcus lactis subsp. cremoris SK11*. Here, the hybrid approach PhymmBL seems to perform better than the (only) composition-based Phymm approach as most of the highly ranked wrong hits are at least in the correct species even if not the correct strain.

| Phymm species | Phymm read count | PhymmBL species | PhymmBL read count |
|---|---|---|---|
| Shewanella amazonensis SB2B | 41000 | Myxococcus xanthus DK 1622 | 132734 |
| Myxococcus xanthus DK 1622 | 40760 | Shewanella amazonensis SB2B | 91286 |
| Lactobacillus brevis ATCC 367 | 31059 | Acidothermus cellulolyticus 11B | 64763 |
| Acidothermus cellulolyticus 11B | 26688 | Lactobacillus brevis ATCC 367 | 54952 |
| Lactobacillus brevis KB290 | 19695 | Lactobacillus casei ATCC 334 | 41734 |
| Lactobacillus casei ATCC 334 | 15668 | Lactobacillus brevis KB290 | 27817 |
| Myxococcus fulvus HW-1 | 13534 | Lactococcus lactis subsp. lactis Il1403 | 9525 |
| Lactobacillus casei str. Zhang | 8154 | Lactobacillus casei str. Zhang | 9396 |
| Corallococcus coralloides DSM 2259 | 6155 | Lactococcus lactis subsp. lactis CV56 | 7056 |
| Myxococcus stipitatus DSM 14675 | 5448 | Lactobacillus casei LC2W | 5083 |
| Lactobacillus casei LC2W | 4385 | Lactobacillus casei W56 | 4209 |
| Lactobacillus casei BL23 | 3926 | Lactobacillus casei BD-II | 4202 |
| Lactobacillus casei BD-II | 3801 | Lactobacillus casei BL23 | 4164 |
| Lactobacillus casei W56 | 3624 | Halobacterium salinarum R1 | 2909 |
| Lactococcus lactis subsp. lactis Il1403 | 3596 | Lactococcus lactis subsp. lactis KF147 | 2882 |
| Lactococcus lactis subsp. lactis CV56 | 3076 | Halobacterium sp. NRC-1 | 2873 |
| Halobacterium salinarum R1 | 2211 | Lactococcus lactis subsp. lactis IO-1 | 1968 |
| Halobacterium sp. NRC-1 | 2180 | Pediococcus pentosaceus ATCC 25745 | 1166 |
| Lactococcus lactis subsp. lactis KF147 | 1779 | Myxococcus fulvus HW-1 | 913 |
| Stigmatella aurantiaca DW4SLASH3-1 | 1770 | Lactococcus lactis subsp. cremoris A76 | 702 |
| Lactococcus lactis subsp. lactis IO-1 | 1764 | Lactobacillus plantarum subsp. plantarum P-8 | 621 |
| Azospirillum brasilense Sp245 | 1119 | Lactobacillus plantarum WCFS1 | 334 |
| Deinococcus gobiensis I-0 | 1113 | Lactobacillus rhamnosus Lc 705 | 275 |
| Lactobacillus plantarum subsp. plantarum P-8 | 1026 | Bacillus cereus FRI-35 | 243 |
| Pseudonocardia dioxanivorans CB1190 | 925 | Lactococcus lactis subsp. cremoris SK11 | 208 |
| Sinorhizobium fredii USDA 257 | 921 | Nonlabens dokdonensis DSW-6 | 200 |
| Kineococcus radiotolerans SRS30216 | 911 | Pediococcus claussenii ATCC BAA-344 | 173 |
| Frankia symbiont of Datisca glomerata | 894 | Lactococcus lactis subsp. cremoris MG1363 | 171 |
| Lactococcus lactis subsp. cremoris A76 | 885 | Bacillus cereus ATCC 10987 | 153 |
| Streptomyces cattleya NRRL 8057 = DSM 46488 | 878 | Bacillus cereus AH187 | 146 |
